# Mean Field Limits for Interacting Diffusions in a Two-Scale Potential

**DOI:** 10.1007/s00332-017-9433-y

**Published:** 2017-12-19

**Authors:** S. N. Gomes, G. A. Pavliotis

**Affiliations:** Department of Mathematics, Imperial College London, London, SW7 2AZ UK

**Keywords:** McKean–Vlasov equation, Interacting particles, Multiscale diffusions, Bifurcation diagram, Phase transitions, Desai–Zwanzig model, Curie–Weiss model, 35Q70, 35Q83, 35Q84, 82B26, 82B80

## Abstract

In this paper, we study the combined mean field and homogenization limits for a system of weakly interacting diffusions moving in a two-scale, locally periodic confining potential, of the form considered in Duncan et al. (Brownian motion in an N-scale periodic potential, arXiv:1605.05854, 2016b). We show that, although the mean field and homogenization limits commute for finite times, they do not, in general, commute in the long time limit. In particular, the bifurcation diagrams for the stationary states can be different depending on the order with which we take the two limits. Furthermore, we construct the bifurcation diagram for the stationary McKean–Vlasov equation in a two-scale potential, before passing to the homogenization limit, and we analyze the effect of the multiple local minima in the confining potential on the number and the stability of stationary solutions.

## Introduction

Systems of interacting particles, possibly subject to thermal noise, arise in several applications, ranging from standard ones such as plasma physics and galactic dynamics (Binney and Tremaine 2008) to dynamical density functional theory (Goddard et al. 2012a, b), mathematical biology (Farkhooi and Stannat 2017; Lućon and Stannat 2016) and even in mathematical models in the social sciences (Garnier et al. 2017; Motsch and Tadmor 2014). As examples of models of interacting “agents” in a noisy environment that appear in the social sciences—which has been the main motivation for this work—we mention the modeling of cooperative behavior (Dawson 1983), risk management (Garnier et al. 2013) and opinion formation (Garnier et al. 2017). Another recent application that has motivated this work is that of global optimization (Pinnau et al. 2017).

In this work, we will consider a system of interacting particles in one dimension, moving in a confining potential, that interact through their mean, i.e., a Curie–Weiss type interaction (Dawson 1983):

1.1 $$\begin{aligned} \hbox {d}X^i_t = \left( -V'(X_t^i) - \theta \left( X_t^i - \frac{1}{N} \sum _{j=1}^N X_t^j \right) \right) \, \hbox {d}t + \sqrt{2 \beta ^{-1}} \, \hbox {d}B_t^i. \end{aligned}$$

Here ${{\mathbf{x}}}_t:= \{ X_t^i \}_{i=1}^N$ denotes the position of the interacting agents, $V(\cdot )$ a confining potential, $\theta$ the strength of the interaction between the agents, $\{ B_t^i \}_{i=1}^N$ standard independent one-dimensional Brownian motions and $\beta$ the inverse temperature. The total energy (Hamiltonian) of the system of interacting diffusions (1.1) is

1.2 $$\begin{aligned} W_N({\mathbf{x}}) = \sum _{\ell =1}^N V(X^{\ell }) + \frac{\theta }{4 N} \sum _{n=1}^N \sum _{\ell =1}^N (X^n - X^{\ell })^2. \end{aligned}$$

Passing rigorously to the mean field limit as $N\rightarrow \infty$ using, for example, martingale techniques (Dawson 1983; Gärtner 1988; Oelschläger 1984), and under appropriate assumptions on the confining potential and on the initial conditions (propagation of chaos), is a well-studied problem. Formally, using the law of large numbers we deduce that

$$\begin{aligned} \lim _{N \rightarrow +\infty } \frac{1}{N} \sum _{j=1}^N X^j_t = {\mathbb {E}}{\mathbf{x}}_t, \end{aligned}$$

where the expectation is taken with respect to the “1-particle” distribution function *p*(*x*, *t*).^1^ Passing, formally, to the limit as $N\rightarrow \infty$ in the stochastic differential equation (1.1), we obtain the McKean SDE

1.3 $$\begin{aligned} d {\mathbf{x}}_t = -V'({ \mathbf{x}}_t) \, \hbox {d}t - \theta ({\mathbf{x}}_t - {\mathbb {E}}{\mathbf{x}}_t) \, \hbox {d}t + \sqrt{2 \beta ^{-1}} \, \hbox {d}B_t. \end{aligned}$$

The Fokker–Planck equation corresponding to this SDE is the McKean–Vlasov equation (Frank 2005; McKean 1966, 1967)

1.4 $$\begin{aligned} \frac{\partial p}{\partial t} = \frac{\partial }{\partial x} \left( V'(x) p + \theta \left( x - \int _{{\mathbb {R}}} x p(x,t) \, \hbox {d}x \right) p + \beta ^{-1} \frac{\partial p}{\partial x} \right) . \end{aligned}$$

The McKean–Vlasov equation is a nonlinear, nonlocal Fokker–Planck type equation that we will sometimes refer to as the McKean–Vlasov–Fokker–Planck equation. It is a gradient flow, with respect to the Wasserstein metric, for the free energy functional

1.5 $$\begin{aligned} {\mathcal {F}}[\rho ] = \beta ^{-1} \int \rho \ln \rho \, \hbox {d}x + \int V \rho \, \hbox {d}x + \frac{\theta }{2} \int \int F(x-y) \rho (x) \rho (y) \, \hbox {d}x \, \hbox {d}y, \end{aligned}$$

where we write the interaction potential as $F(x) = \frac{1}{2} x^2$. Background material on the McKean–Vlasov equation can be found in, e.g., Carrillo et al. (2006), Frank (2005) and Villani (2003).

The finite-dimensional dynamics (1.1) has a unique invariant measure. Indeed, the process ${\mathbf{x}}_t$ defined in (1.1) with *V* being a confining potential is always ergodic, and in fact reversible, with respect to the Gibbs measure (Pavliotis 2014, Ch. 4),

1.6 $$\begin{aligned} \mu _N (\hbox {d}{\mathbf{x}}) = \frac{1}{Z_N} e^{-\beta W_N({\mathbf{x}^1, \dots \mathbf{x}^N})} \, \hbox {d}x^1 \dots \hbox {d}{\mathbf{x}}^N, \quad Z_N = \int _{{\mathbb {R}}^N} e^{-\beta W_N({\mathbf{x}^1, \dots \mathbf{x}^N})} \, \hbox {d}{\mathbf{x}^1 \dots \hbox {d}\mathbf{x}^N} \end{aligned}$$

where $W_N(\cdot )$ is given by (1.2).

On the other hand, the McKean dynamics (1.3) and the corresponding McKean–Vlasov–Fokker–Planck equation (1.4) can have more than one invariant measures, for nonconvex confining potentials and at sufficiently low temperatures (Dawson 1983; Tamura 1984). This is not surprising, since the McKean–Vlasov equation is a nonlinear, nonlocal PDE and the standard uniqueness of solutions for the linear (stationary) Fokker–Planck equation does not apply (Bogachev et al. 2015).

The density of the invariant measure(s) for the McKean dynamics (1.3) satisfies the stationary nonlinear Fokker–Planck equation

1.7 $$\begin{aligned} \frac{\partial }{\partial x} \left( V'(x) p_{\infty } + \theta \left( x - \int _{{\mathbb {R}}} x p_{\infty }(x) \, \hbox {d}x \right) p_{\infty } + \beta ^{-1} \frac{\partial p_{\infty }}{\partial x} \right) =0. \end{aligned}$$

Based on earlier work (Dawson 1983; Tamura 1984), it is by now well understood that the number of invariant measures, i.e., the number of solutions to (1.7), is related to the number of metastable states (local minima) of the confining potential—see Tugaut (2014) and the references therein.

For the Curie–Weiss (i.e., quadratic) interaction potential a one-parameter family of solutions to the stationary McKean–Vlasov equation (1.7) can be obtained:

1.8a $$\begin{aligned} p_{\infty }(x ; \theta , \beta , m)= & {} \frac{1}{Z(\theta , \beta ; m )} e^{- \beta \left( V(x) + \theta \left( \frac{1}{2}x^2 - x m \right) \right) }, \end{aligned}$$

1.8b $$\begin{aligned} Z(\theta , \beta ; m )= & {} \int _{{\mathbb {R}}} e^{- \beta \left( V(x) + \theta \left( \frac{1}{2}x^2 - x m \right) \right) } \, \hbox {d}x. \end{aligned}$$

This one-parameter family of probability densities is subject, of course, to the constraint that it provides us with the correct formula for the first moment:

1.9 $$\begin{aligned} m = \int _{{\mathbb {R}}} x p_{\infty }(x ; \theta , \beta , m) \, \hbox {d}x =: R(m; \theta , \beta ). \end{aligned}$$

We will refer to this as the **self-consistency** equation and it will be the main object of study of this paper. Once a solution to (1.9) has been obtained, substitution back into (1.8) yields a formula for the invariant density $p_{\infty }(x ; \theta , \beta , m)$.

**Fig. 1:**
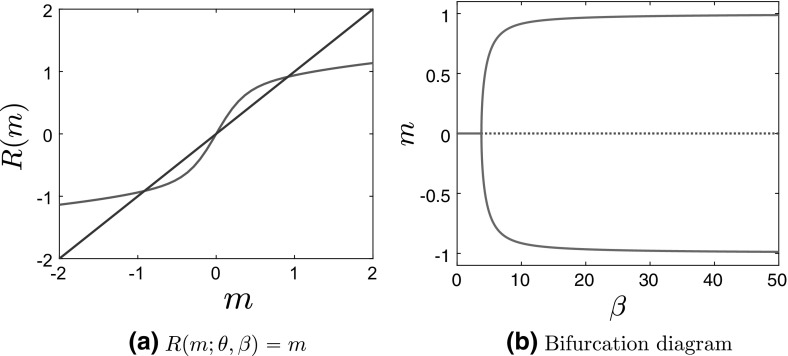
**a** Plot of $R(m;\theta ,\beta )$ and of the straight line $y =x$ for $\theta = 0.5$, $\beta = 10$, and **b** bifurcation diagram of *m* as a function of $\beta$ for $\theta = 0.5$ for the bistable potential $V(x) = \frac{x^4}{4}-\frac{x^2}{2}$ and interaction potential $F(x) = \frac{x^2}{2}$

Clearly, the number of invariant measures of the McKean–Vlasov dynamics is determined by the number of solutions to the self-consistency equation (1.9). It is well known and not difficult to prove that for symmetric nonconvex confining potentials a unique invariant measure exists at sufficiently high temperatures, whereas more than one invariant measure exists below a critical temperature $\beta ^{-1}_c$ (Dawson 1983, Thm. 3.3.2; Tamura 1984, Thm. 4.1, Thm. 4.2); see also Shiino (1987). In particular, for symmetric potentials, $m = 0$ is always a solution to the self-consistency equation (1.9). Above $\beta _c$, i.e., at sufficiently low temperatures, the zero solution loses stability and a new branch bifurcates from the $m = 0$ solution (Shiino 1987). This second-order phase transition is similar to the one familiar from the theory of magnetization and the study of the Ising model. In Fig. 1, we present the solution to the self-consistency equation and the bifurcation diagram for stationary solutions of the McKean–Vlasov equation for the standard bistable—Landau—potential $V(x) = \frac{x^4}{4}-\frac{x^2}{2}$.

To compute the critical temperature, we need to solve the equation obtained by differentiating the self-consistency equation with respect to the order parameter *m* at $m=0$ (see Shiino 1987; Frank 2005, Sec 5.1.3 for more details):

1.10 $$\begin{aligned} {\text {Var}}_{p_{\infty }}(x)\Big |_{m=0}:= & {} \int x^2 p_\infty (x; \beta , \theta ,m=0) \ \hbox {d}x - \left( \int x p_\infty (x;\beta , \theta ,m=0) \ \hbox {d}x )\right) ^2\nonumber \\= & {} \frac{1}{\beta \theta }. \end{aligned}$$

The number of times that *m* and $R(m;\theta ,\beta )$ cross, i.e., the number of stationary measures, depends on the slope of $R(m;\theta ,\beta )$ at the origin. This is given precisely by Eq. (1.10).

The main purpose of this paper is to study the dynamics and, in particular, bifurcations and phase transitions for a system of interacting diffusions moving in a rugged energy landscape, coupled through the Curie–Weiss interaction. We are particularly interested in understanding the combined effect of the presence of several local minima (metastable states) in the confining potential and of the passage to the mean field limit. We will study the problem for a system of interacting diffusions of the form (1.1) moving in a two-scale, locally periodic confining potential

1.11 $$\begin{aligned} V^{\epsilon }(\mathbf{x}) = V\left( \mathbf{x}, \frac{\mathbf{x}}{\epsilon } \right) , \end{aligned}$$

where $V: \, (\mathbf{x},\mathbf{y})\in {\mathbb {R}}\times \mathcal {Y}\rightarrow {\mathbb {R}}$, $\mathcal {Y}$ denotes a periodic box in ${\mathbb {R}}^d$, $\mathcal {Y}= [0,L]^d$:

1.12 $$\begin{aligned} V(\mathbf{x},\mathbf{y}+kLe_i) = V(\mathbf{x},\mathbf{y}), \quad k\in \mathbb {Z}, \end{aligned}$$

and $\left\{ e_1,\dots ,e_d\right\}$ is the canonical basis of ${\mathbb {R}}^d$. Throughout this paper, $L = 2\pi$. The particles $\{ X_t^i, \, i=1,\dots ,N \}$ are interacting through the Curie–Weiss interaction, $F(x) = \frac{x^2}{2}$. This class of potentials provides us with a natural testbed for testing several techniques and methodologies for the study of multiscale diffusions such as maximum likelihood estimation (Papavasiliou et al. 2009; Pavliotis and Stuart 2007), particle filters and filtering (Imkeller et al. 2013; Papavasiliou 2007), importance sampling and large deviations (Spiliopoulos 2013) and optimal control (Hartmann et al. 2014).

Of particular relevance to us is the multiscale analysis presented in Duncan et al. (2016a), Duncan et al. (2016b).^2^ In these works, the homogenized SDE for a Brownian particle moving in a two-scale potential in ${\mathbb {R}}^d$ valid in the limit of infinite scale separation $\epsilon \rightarrow 0$ was obtained and the effect of the multiscale structure on noise-induced transitions was investigated. It was shown, in particular, that the homogenized SDE is characterized by multiplicative noise. For a single Brownian particle in ${\mathbb {R}}^d$ moving in a two-scale potential (1.11) (or, equivalently, for a system of *d* noninteracting Brownian particles in a two-scale potential), the homogenized equation reads

1.13 $$\begin{aligned} \hbox {d}X_t = -{\mathcal {M}}(X_t) \nabla \Psi (X_t) \, \hbox {d}t + \beta ^{-1}(\nabla \cdot {\mathcal {M}})(X_t) \, \hbox {d}t + \sqrt{2 \beta ^{-1}{\mathcal {M}}(X_t)} \, \hbox {d}B_t, \end{aligned}$$

where ${\mathcal {M}}(\cdot )$ denotes the diffusion tensor and $\Psi (\cdot )$ the free energy—see Sect. 2. It is important to note that, in addition to the presence of multiplicative noise, the potential energy driving the dynamics is not simply the average of the two-scale potential over its period, but, rather, the free energy $\Psi = - \beta ^{-1} \ln \left( \int e^{-\beta V(x,y)} \, \hbox {d}y \right)$. Since the dynamics (1.13) is finite-dimensional, no phase transitions can occur. In fact, the homogenized dynamics is reversible with respect to the thermodynamically consistent Gibbs measure; see the discussion in Sect. 2. It is well known, however, that multiplicative noise can lead to noise-induced transitions, i.e., to changes in the topological structure of the invariant measure (Horsthemke and Lefever 1984; Pavliotis 2014, Sec. 5.4). Such phenomena, including multiscale-induced hysteresis effects, for a one-dimensional Brownian particle moving in a multiscale potential, were studied in detail in Duncan et al. (2016a).

Our goal is to study mean field limits for multiscale interacting diffusions of the form

1.14 $$\begin{aligned} \hbox {d}X^{\epsilon ,i}_t = -\nabla V^\epsilon (X_t^{\epsilon ,i})\,\hbox {d}t - \frac{\theta }{N}\sum _{j=1}^{N} \nabla F(X_t^{\epsilon ,i} - X_t^{\epsilon ,j})\,\hbox {d}t + \sqrt{2\beta ^{-1}}\hbox {d}B_t^i, \end{aligned}$$

where the two-scale potential is given by (1.11). The interaction potential $F(\cdot )$ is assumed to be a smooth even function, with $F(0) = 0$ and $F'(0)= 0$. All of the numerical experiments that we will present will be for the Curie–Weiss quadratic interaction potential $F(x) = \frac{1}{2}x^2$.

The main issues that we address in this work are:

1. What is the effect of the presence of (infinitely) many local minima in the locally periodic confining potential on the bifurcation diagram? In other words, how do the bifurcation diagrams for $\epsilon \ll 1$ but finite and $\epsilon \rightarrow 0$ differ?
2. Do the homogenization and mean field limits commute, in particular when also passing to the long time limit $T \rightarrow +\infty$? In other words: are the bifurcations diagrams corresponding to the $N\rightarrow \infty , \, T\rightarrow \infty , \, \epsilon \rightarrow 0$ and $\epsilon \rightarrow 0, \, N\rightarrow \infty , \, T\rightarrow \infty$ limits the same?

**Fig. 2:**
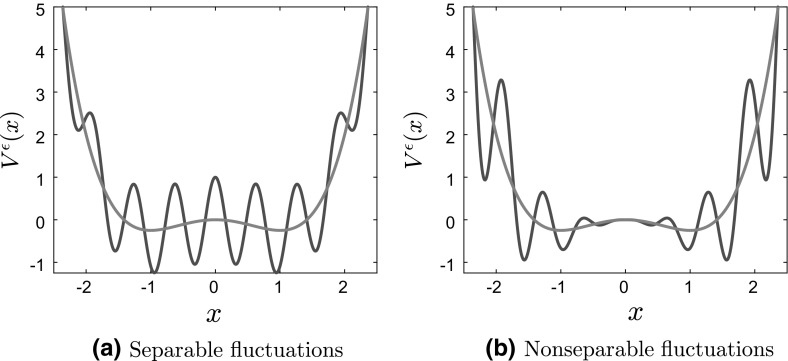
Bistable potential with (left) separable and (right) nonseparable fluctuations

Two typical examples of the type of locally periodic potentials that we will study in this paper are shown in Fig. 2:

1.15 $$\begin{aligned} V^{\epsilon }(x) = \frac{x^4}{4} - \frac{x^2}{2} + \delta \cos \left( \frac{x}{\epsilon } \right) \quad \text{ and } \quad V^{\epsilon }(x) = \frac{x^4}{4} - \left( 1 - \delta \cos \left( \frac{x}{\epsilon } \right) \right) \frac{x^2}{2}. \end{aligned}$$

It should be clear from these two figures that the homogenization and mean field limits, when also combined with the long time limit, do not necessarily commute. First, the homogenization process tends to smooth out local minima and to even “convexify” the confining potential—think of a quadratic potential perturbed by fast periodic fluctuations. This implies, in particular, that even though many additional stationary solutions, i.e., branches in the bifurcation diagram may appear for all finite values of $\epsilon$, most, if not all, of them may not be present in the bifurcation diagram for the homogenized dynamics. Furthermore, multiplicative/nonseparable fluctuations of the type presented in Fig. 2b tend to flatten the potential around $x=0$. As we will see in Sect. 4, this phenomenon is very much related to the lack of commutativity of the limits $N\rightarrow \infty , \, T\rightarrow \infty , \, \epsilon \rightarrow 0$ and $\epsilon \rightarrow 0, \, N\rightarrow \infty , \, T\rightarrow \infty$.

We will study these problems using a combination of formal multiscale calculations, (some) rigorous analysis and extensive numerical simulations. There are many technical issues that we do not address, such as the rigorous homogenization study of the McKean–Vlasov equation and the rigorous study of bifurcations in the presence of infinitely many local minima. We will address these in future work.

The rest of the paper is organized as follows. In Sect. 2, we study the mean field limit for a system of homogenized interacting diffusions, i.e., the first $\epsilon \rightarrow 0$, then $N\rightarrow \infty$ limit. In Sect. 3, we study the homogenization problem for the McKean–Vlasov equation in a two-scale potential. In Sect. 4, we present extensive numerical simulations. Section 5 is reserved for conclusions.

## Mean Field Limit of the Homogenized Interacting Diffusions: First $\epsilon \rightarrow 0$, then $N\rightarrow \infty$

In this section, we consider the one-dimensional version of the system of SDEs (1.14). We first take the homogenization limit ($\epsilon \rightarrow 0$) and then the mean field limit ($N\rightarrow \infty$). The homogenization theorem for a system of finite-dimensional interacting diffusions moving in a two-scale confining potential is presented in Duncan et al. (2016b). The mean field limit of the homogenized SDE system can be obtained by using the results of Gärtner (1988), Oelschläger (1984).

### Homogenization for Finite System of Interacting Diffusions in a Two-Scale Potential

We consider the system of interacting diffusions

2.1 $$\begin{aligned} \hbox {d}X_t^i = -\partial _x V^\epsilon (X_t^i)\,\hbox {d}t - \frac{\theta }{N}\sum _{j=1}^{N} \partial _x F(X_t^i - X_t^j)\,\hbox {d}t + \sqrt{2\beta ^{-1}}\hbox {d}B_t^i, \end{aligned}$$

where *F* is a smooth even function with $F(0) = 0$ and $F'(0)= 0$ and $V^\epsilon$ is a smooth locally periodic potential of the form (2.7). We introduce the notation $\mathbf{x}_t = (X_t^1, \dots , X_t^N)$, so that we have

2.2 $$\begin{aligned} \hbox {d}\mathbf{x}_t^\epsilon = -\nabla W^\epsilon (\mathbf{x}_t^\epsilon ) \, \hbox {d}t +\sqrt{2\beta ^{-1}}\hbox {d}B_t, \end{aligned}$$

where

$$\begin{aligned} W^\epsilon (\mathbf{x}) = \sum _{\ell =1}^N V^\epsilon \left( \mathbf{x}^{\ell },\frac{\mathbf{x}^\ell }{\epsilon }\right) + \frac{\theta }{{2} N} \sum _{n=1}^N \sum _{\ell =1}^N F(\mathbf{x}^n - \mathbf{x}^{\ell }). \end{aligned}$$

and $B_t$ is a standard Brownian motion in ${\mathbb {R}}^N$. This equation is of the same form as Duncan et al. 2016b, Eq. (1) and Duncan et al. (2016a), Eqn.(1), with $V^\epsilon (X_t)$ replaced by $W^\epsilon (\mathbf{x}_t^\epsilon )$.

Since *F* does not depend on the fast scale, the results of Duncan et al. (2016b) apply directly to (2.2) and we deduce that the sequence $\left\{ \mathbf{x}_t^\epsilon \right\}$ converges, as $\epsilon \rightarrow 0$, to the solution of the homogenized equation

2.3 $$\begin{aligned} \hbox {d}\mathbf{x}_t = -\left[ {\mathcal {M}}(\mathbf{x}_t)\nabla \Psi _N(\mathbf{x}_t) - \beta ^{-1}\nabla \cdot {\mathcal {M}}(\mathbf{x}_t)\right] \, \hbox {d}t + \sqrt{2\beta ^{-1}{\mathcal {M}}(\mathbf{x}_t)}\hbox {d}B_t, \end{aligned}$$

where

2.4 $$\begin{aligned} \Psi _N (\mathbf{x}) = -\beta ^{-1}\ln {\mathcal {Z}}_N(\mathbf{x}), \end{aligned}$$

for

2.5 $$\begin{aligned} {\mathcal {Z}}_N(\mathbf{x}) = \int _{\mathcal {Y}} e^{-\beta W_N(\mathbf{x},\mathbf{y})} \ \hbox {d}\mathbf{y}, \end{aligned}$$

where $W_N(\mathbf{x},\mathbf{y})$ is defined as in Eq. (1.2),

2.6 $$\begin{aligned} W_N(\mathbf{x},\mathbf{y}) = \sum _{\ell =1}^N V(\mathbf{x}^{\ell },\mathbf{y}^\ell ) + \frac{\theta }{{2} N} \sum _{n=1}^N \sum _{\ell =1}^N F(\mathbf{x}^n - \mathbf{x}^{\ell }). \end{aligned}$$

The convergence is in the sense of weak convergence of probability measures, i.e., the law of the process $\mathbf{x}_t^\epsilon$ converges weakly to the law of the limiting process $\mathbf{x}_t$. The proof of this result, which is quite standard, is based on the application of Itô’s formula to the solution of an appropriate Poisson equation, Eq. (2.12), the decomposition of the rescaled process into a martingale part and a remainder part, and the use of the martingale central limit theorem. The details can be found in Duncan et al. (2016b). It will be useful to decompose the two-scale potential into its large-scale confining part and the modulated, mean-zero, fluctuations:

2.7 $$\begin{aligned} V^\epsilon (x) = V_0(x) + V_1 \left( x,\frac{x}{\epsilon } \right) , \quad V_0(x) = \int _{\mathcal {Y}} V(x, y) \, \hbox {d}y. \end{aligned}$$

Notice that this decomposition is not unique, since we can define the average of the two-scale potential over the unit cell with respect to a different, e.g., Gibbs, weight. However, the choice of the weight does not affect our results. See, e.g., the proof of Proposition 3.1.

We note that the free energy $\Psi _N$ is of the form

2.8 $$\begin{aligned} \Psi _N(\mathbf{x}) = \left( \sum _{\ell =1}^N V_0(x^{\ell }) + \frac{\theta }{{2} N} \sum _{n=1}^N \sum _{\ell =1}^N F(x^n - x^{\ell }) \right) +\psi (\mathbf{x}), \end{aligned}$$

where

2.9 $$\begin{aligned} \psi (\mathbf{x})&= -\beta ^{-1}\ln \left( \prod _{\ell =1}^N \int _{\mathcal {Y}} e^{-\beta V_1(x^{\ell },y^{\ell })} \ \hbox {d}y^{\ell }\right) \nonumber \\&= -\beta ^{-1}\sum _{\ell =1}^N \ln \left( \int _{\mathcal {Y}} e^{-\beta V_1(x^{\ell },y)} \ \hbox {d}y\right) . \end{aligned}$$

Finally, ${\mathcal {M}}:{\mathbb {R}}^{d}\rightarrow {\mathbb {R}}^{d\times d}_{sym}$ is defined by

2.10 $$\begin{aligned} {\mathcal {M}}(\mathbf{x}) = \frac{{\mathcal {K}}(\mathbf{x})}{{\mathcal {Z}}_N(\mathbf{x})}, \end{aligned}$$

where

2.11 $$\begin{aligned} {\mathcal {K}}(\mathbf{x}) = \int _{\mathcal {Y}} (I + \nabla _y \Phi (\mathbf{x}, \mathbf{y}))e^{-\beta W_N(\mathbf{x},\mathbf{y})}\,\hbox {d}\mathbf{y},\quad \mathbf{x}\in {\mathbb {R}}^d, \end{aligned}$$

and, for fixed $\mathbf{x}\in {\mathbb {R}}^d$, $\Phi$ is the unique weak solution in $H^1(\mathcal {Y})$ to

2.12 $$\begin{aligned} \nabla _\mathbf{y}\cdot \left( e^{-\beta V_1(\mathbf{x},\mathbf{y})}(I + \nabla _\mathbf{y}\Phi (\mathbf{x},\mathbf{y}))\right) = 0,\quad \mathbf{y}\in \mathcal {Y}, \end{aligned}$$

or

$$\begin{aligned} \sum _{i=1}^N \frac{\partial }{\partial y^i}\left( e^{-\beta V_1(\mathbf{x},\mathbf{y})}\left( \delta _{ij} + \frac{\partial \Phi _j(\mathbf{x},\mathbf{y})}{\partial y^i}\right) \right) = 0,\quad j=1,\dots ,N, \end{aligned}$$

such that the centering condition $\int _\mathcal {Y}\Phi (\mathbf{x},\mathbf{y})e^{-\beta V_1(\mathbf{x},\mathbf{y})}\,\hbox {d}\mathbf{y}= 0$, for all $\mathbf{x}\in {\mathbb {R}}^d$ is satisfied. The proof of uniqueness of centered solutions to this equation is based on the Lax–Milgram lemma and can be found in Duncan and Pavliotis (2016b, Thm. 2.3).

To compute the diffusion tensor (see Duncan et al. 2016a, Appendix A for a similar computation), we observe that

$$\begin{aligned} {{\mathcal {M}}_{ij}}(\mathbf{x})= & {} \delta _{ij} + \frac{1}{{{\mathcal {Z}}_N(\mathbf{x})}}\\&\,\, \times \int _{\mathcal {Y}}\frac{\partial \Phi _i}{\partial y^j}(\mathbf{x},\mathbf{y})e^{-\beta \left( \sum _{\ell =1}^N V_0(x^\ell )+\sum _{\ell =1}^N V_1(x^\ell ,y^\ell )+{\frac{\theta }{2N} \sum _{n=1}^N\sum _{\ell =1}^N F(x^\ell -x^n)}\right) } \ \hbox {d}\mathbf{y}\\= & {} \delta _{ij} + \frac{1}{\bar{{\mathcal {Z}}}(\mathbf{x})} \int _0^L \cdots \int _0^L \frac{\partial \Phi _i}{\partial y^j}(\mathbf{x},\mathbf{y})\prod _{m=1}^N e^{-\beta V_1(x^m,y^m)} \ \hbox {d}y^m, \end{aligned}$$

where ${\mathcal {Z}}(\mathbf{x})$ is defined in Eq. (2.5) and

2.13 $$\begin{aligned} \bar{{\mathcal {Z}}}(\mathbf{x}) = \prod _{m=1}^N \int _0^L e^{-\beta V_1(x^m,y^m)} \ \hbox {d}y^m. \end{aligned}$$

Since there is no coupling between the different $y^i$ components od Eq. (2.12), it follows that $\Phi (\mathbf{x},\mathbf{y})$ can be written in the form $\Phi (\mathbf{x},\mathbf{y}) = (\phi (x^1,y^1),\phi (x^2,y^2),\dots ,\phi (x^N,y^N))$, where $\phi (x,y)$ solves

2.14 $$\begin{aligned} -{\mathcal {L}}_0\phi (x,y) = -\frac{\partial V_1}{\partial y}(x,y), \qquad {\mathcal {L}}_0 = -\partial _y V_1 \partial _y +\beta ^{-1}\partial ^2_y, \end{aligned}$$

and therefore $\Phi _i(x,y) = \phi (x^i,y^i)$ and

$$\begin{aligned} \frac{\partial \Phi _i}{\partial y^j}(x,y) = \frac{\partial \phi (x^i,y^i)}{\partial y^j} = \delta _{ij}\frac{\partial \phi }{\partial y^j}(x^i,y^i). \end{aligned}$$

Substituting in (2.13), we obtain

2.15 $$\begin{aligned} {{\mathcal {M}}}_{ij}(\mathbf{x}) = \displaystyle {=\delta _{ij} + \frac{1}{\bar{{\mathcal {Z}}}(\mathbf{x})} \int _0^L \cdots \int _0^L \delta _{ij} \frac{\partial \phi }{\partial y^j}(x^i,y^i)\prod _{m=1}^N e^{-\beta V_1(x^m,y^m)} \ \hbox {d}y^m,} \end{aligned}$$

and the diffusion tensor is diagonal, with

$$\begin{aligned} {{\mathcal {M}}}_{ii}(\mathbf{x})= & {} 1 + \frac{1}{\prod _{m=1}^N \int _0^L e^{-\beta V_1(x^m,y^m)} \ \hbox {d}y^m} \left( \int _0^L \frac{\partial \phi }{\partial y^i}(x^i,y^i) e^{-\beta V_1(x^i,y^i)} \ \hbox {d}y^i\right) \\&\times \int _0^L \prod _{m=1, m\ne i}^N e^{-\beta V_1(x^m,y^m)} \ \hbox {d}y^m \\= & {} \displaystyle {1 + \frac{1}{ \int _0^L e^{-\beta V_1(x^i,y^i)} \ \hbox {d}y^i} \int _0^L \frac{\partial \phi }{\partial y^i}(x^i,y^i) e^{-\beta V_1(x^i,y^i)} \ \hbox {d}y^i.} \end{aligned}$$

As it is well known (Pavliotis and Stuart 2008, Sec 13.6.1), the one-dimensional Poisson equation (2.14) can be solved explicitly, up to quadratures. We can then obtain formulas for the diagonal elements ${\mathcal {M}}_{ii}$ of the diffusion tensor ${\mathcal {M}}(x)$:

2.16 $$\begin{aligned} {\mathcal {M}}(x) = \frac{1}{\left( \frac{1}{L}\int _0^L e^{-\beta V_1(x,y)} \, \hbox {d}y\right) \left( \frac{1}{L}\int _0^L e^{\beta V_1(x,y)} \, \hbox {d}y\right) }. \end{aligned}$$

We can write the system of stochastic differential equations for the homogenized system of interacting particles:

2.17 $$\begin{aligned} \hbox {d}X_t^i = -\left[ {\mathcal {M}}(X_t^i) \partial _{x^i}\Psi (X_t^1, \dots X_t^N) - \beta ^{-1}{\mathcal {M}}'(X_t^i)\right] \, \hbox {d}t + \sqrt{2\beta ^{-1}{\mathcal {M}}(X_t^i)}\hbox {d}B_t^i, \end{aligned}$$

for $i=1,\dots ,N$, where ${\mathcal {M}}$ is defined in Eq. (2.16), prime denotes differentiation with respect to *x* and $\Psi$ is given by Equations (2.8)-(2.9).

We note that the homogenized system of SDEs (2.17) is characterized by multiplicative noise.^3^ Furthermore, the diffusion coefficient of the *i*th particle depends only on the position of the particle itself, and not of the other particles. The dynamics (2.17) is reversible with respect to the Gibbs measure

2.18 $$\begin{aligned} p_{\infty }(\hbox {d}x) = \frac{1}{\bar{Z}} e^{-\beta \Psi (x)} \, \hbox {d}x, \quad \bar{Z} = \int _{\mathbb {R}}e^{-\beta \Psi (x)} \ \hbox {d}x. \end{aligned}$$

### Mean Field Limit for the Homogenized SDE

We can now pass to the mean field limit $N\rightarrow \infty$. The system of SDEs (2.17) is of the form

$$\begin{aligned} \hbox {d}X_t^i = b\left( X_t^i,\frac{1}{N}\sum _{j=1}^N X_t^j\right) \hbox {d}t + \sigma (X_t^i) \hbox {d}B_t^i, \end{aligned}$$

which is in the same form to the one considered in Gärtner (1988), Oelschläger (1984), with slightly different drift and diffusion coefficients.^4^ It is straightforward to check that the homogenized equation satisfies the conditions in the aforementioned papers.^5^ Taking the mean field limit of (2.17), we obtain the following nonlinear Fokker–Planck equation:

2.19 $$\begin{aligned} \frac{\partial p}{\partial t}= & {} \frac{\partial }{\partial x}\left[ \beta ^{-1}\frac{\partial \left( {\mathcal {M}}(x) p\right) }{\partial x} + {\mathcal {M}}(x)\left( V'_0(x) +\psi '(x) + \theta \left( F' \star p\right) (x)\right) p\right. \nonumber \\&\left. + \,\beta ^{-1}\frac{\partial {\mathcal {M}}(x)}{\partial x}p \right] , \end{aligned}$$

where $\star$ denotes the convolution operator in *x*,

2.20 $$\begin{aligned} \psi (x) = -\beta ^{-1}\ln \left( \int _0^L e^{-\beta V_1(x,y)} \ \hbox {d}y \right) , \end{aligned}$$

and ${\mathcal {M}}(x)$ is defined in (2.10). We note that the solution of Eq. (2.19) represents the density of the empirical measure of the process in the limit $N\rightarrow \infty$.

The McKean stochastic differential equation corresponding to (2.19) is

2.21 $$\begin{aligned} \hbox {d}X_t= & {} -\,{\mathcal {M}}(X_t) (V'_0(X_t) + \psi '(X_t) +{\frac{\theta }{N} \sum _{\ell =1}^N F'(X_t-X_t^\ell )}) \, \hbox {d}t\nonumber \\&+\, \beta ^{-1} {\mathcal {M}}'(X_t) \, \hbox {d}t + \sqrt{2 \beta ^{-1} {\mathcal {M}}(X_t)} \, \hbox {d}B_t. \end{aligned}$$

We reiterate that the correction to the drift $\beta ^{-1} {\mathcal {M}}'(X_t) \, \hbox {d}t$ is not the Stratonovich correction, but rather the Klimontovich (kinetic) one. This interpretation of the stochastic integral ensures that the homogenized dynamics is reversible with respect to the (thermodynamically consistent) Gibbs measure(s) that we can calculate by solving the stationary Fokker–Planck equation.

The (one or more) stationary distributions $p_\infty (x;\theta , \beta ,m)$ are solutions to the stationary Fokker–Planck equation

2.22 $$\begin{aligned} \mathcal {L}^* p_{\infty }:= & {} \frac{\partial }{\partial x}\left( {\mathcal {M}}(x)\left( V'_0(x) + \psi '(x) + {\theta }(F'\star p_\infty ) p_\infty + \beta ^{-1}p_\infty \right) \right. \nonumber \\&\left. + \,\beta ^{-1}\frac{\partial ({\mathcal {M}}(x)p_\infty )}{\partial x}\right) = 0. \end{aligned}$$

The detailed balance condition implies that

$$\begin{aligned} \beta ^{-1}{\mathcal {M}}(x)\frac{\partial p_\infty }{\partial x} = -{\mathcal {M}}(x)\left( V'_0(x)+ {\theta }(F'\star p_\infty )(x) + \psi '(x)\right) p_\infty , \end{aligned}$$

and since ${\mathcal {M}}(x)$ is strictly positive, a simple variant of Tamura (1984, Lemma 4.1) enables us to obtain an integral equation for the invariant distribution:

2.23 $$\begin{aligned} p_\infty (x;\theta ,\beta ,m)= & {} \frac{1}{Z}e^{-\beta \left( V_0(x)+\theta (F\star p_\infty )(x)+\psi (x)\right) },\nonumber \\ \quad Z= & {} \int _{\mathbb {R}}e^{-\beta \left( V_0(x)+\theta (F\star p_\infty )(x)+\psi (x)\right) } \ \hbox {d}x, \end{aligned}$$

where $\psi (x)$ is given by Eq. (2.20). In particular, $p_{\infty }$ is independent of the diffusion tensor ${\mathcal {M}}(x)$.

For the particular case of a quadratic interaction potential $F(x) = \frac{x^2}{2}$, which is the case that we will study here, all stationary solutions are given by the one-parameter family of Gibbs states of the form (1.8) and the integral equation (2.23) reduces to a nonlinear equation, the self-consistency equation (Shiino 1987)

2.24 $$\begin{aligned} m = R(m;\theta ,\beta ) := \frac{1}{Z}\int _{\mathbb {R}}x e^{-\beta \left( V_0(x)+\theta \left( \frac{x^2}{2}-m x\right) +\psi (x)\right) } \ \hbox {d}x. \end{aligned}$$

By solving this equation, we can construct the full bifurcation diagram of the stationary Fokker–Planck equation. This will be done in Sect. 4.

We are also interested in the equation for the critical temperature (1.10), which in this case is given by

2.25 $$\begin{aligned} \frac{1}{Z}\int _{\mathbb {R}}x^2 e^{-\beta \left( V_0(x)+\theta \left( \frac{x^2}{2}\right) +\psi (x)\right) }\ \hbox {d}x -\left( \frac{1}{Z}\int _{\mathbb {R}}x e^{-\beta \left( V_0(x)+\theta \left( \frac{x^2}{2} \right) +\psi (x)\right) } \ \hbox {d}x\right) ^2= \frac{1}{\beta \theta }. \end{aligned}$$

Assuming that the large-scale part of the potential is symmetric, we have that $\int x p_\infty (x; \beta , \theta ,m=0) \ \hbox {d}x ) = 0$ and the equation above simplifies to

2.26 $$\begin{aligned} \frac{1}{Z} \int _{\mathbb {R}}x^2 e^{-\beta \left( V_0(x)+\theta \left( \frac{x^2}{2}\right) +\psi (x)\right) }\ \hbox {d}x= \frac{1}{\beta \theta }. \end{aligned}$$

From the definition of $\psi (x)$ in Eq. (2.20)), we can conclude that for separable potentials, i.e., when $V_1(x,y)$ is independent of *x*, then $\psi (x)$ becomes a constant. This, in turn, means that the stationary solutions to the homogenized McKean–Vlasov equation are the same to the ones for the system without fluctuations ($V_1(x,y)=0$)—see Corollary 3.2 in Sect. 3. For example, when the large-scale part of the potential $V_0(x)$ is convex, there are no phase transitions for the homogenized dynamics. We will show in Sections 3 and 4 that this is not the case if we take the limits in different order.

## Multiscale Analysis for the McKean–Vlasov Equation in a Two-scale Potential

In this section, we consider the homogenization problem for the McKean–Vlasov equation in a locally periodic potential for the case of a quadratic (Curie–Weiss) interaction. In particular, we first pass to the mean field limit (i.e., send $N\rightarrow \infty$) in Eq. (1.14) with $F(x) = \frac{x^2}{2}$ and study the effects of finite (but small) $\epsilon$ on the bifurcation diagram, before sending $\epsilon \rightarrow 0$.

### Mean Field Limit for Interacting Diffusions in a Two-Scale Potential: $N\rightarrow \infty$, $\epsilon >0$ Finite

We start with the system of interacting diffusions

3.1 $$\begin{aligned} \hbox {d}X_t^i = -\partial _x V \left( X_t^i, \frac{X_t^i}{\epsilon } \right) \,\hbox {d}t - \theta \left( X_t^i - \frac{1}{N} \sum _{j=1}^{N} X_t^j \right) \,\hbox {d}t + \sqrt{2\beta ^{-1}} \, \hbox {d}B_t^i. \end{aligned}$$

The notation is the same as in Sect. 2, i.e., $V^{\epsilon }(x):=V \left( x,\frac{x}{\epsilon } \right)$ is a smooth confining potential that is *L*-periodic in its second argument, $\theta >0$ is the interaction strength, $\beta$ the inverse temperature and $\{ B^i_t, \; i=1, \dots , N \}$ are standard independent one-dimensional Brownian motions.

Taking the limit as $N\rightarrow \infty$, we obtain the McKean–Vlasov–Fokker–Planck equation:

3.2 $$\begin{aligned} \frac{\partial p}{\partial t} = \frac{\partial }{\partial x}\left( \beta ^{-1}\frac{\partial p}{\partial x} + \partial _x V^\epsilon (x) p + \theta \left( x - \int x p(x,t) \, \hbox {d}x \right) p\right) . \end{aligned}$$

The equilibrium solutions, i.e., stationary states, of this equation are given by a one-parameter family of two-scale Gibbs distributions—see Eq. (1.8):

3.3a $$\begin{aligned} p^{\epsilon }_{\infty }(x ; \theta , \beta , m^{\epsilon })= & {} \frac{1}{Z^{\epsilon }(\theta , \beta ; m^{\epsilon } )} e^{- \beta \left( V^{\epsilon }(x) + \theta \left( \frac{1}{2}x^2 - x m^{\epsilon } \right) \right) }, \end{aligned}$$

3.3b $$\begin{aligned} Z^{\epsilon }(\theta , \beta ; m^{\epsilon } )= & {} \int _{{\mathbb {R}}} e^{- \beta \left( V^{\epsilon }(x) + \theta \left( \frac{1}{2}x^2 - x m^{\epsilon } \right) \right) } \, \hbox {d}x. \end{aligned}$$

Our goal now is to study the $\epsilon \rightarrow 0$ limit of the self-consistency equation—see Eq. (1.9)

3.4 $$\begin{aligned} m^{\epsilon } = \int _{{\mathbb {R}}} x p^{\epsilon }_{\infty }(x ; \theta , \beta , m^{\epsilon }) \, \hbox {d}x =: R^{\epsilon } (m^{\epsilon }; \theta , \beta ), \end{aligned}$$

and also the equation for the critical temperature,

3.5 $$\begin{aligned} \int _{{\mathbb {R}}} x^2 p^{\epsilon }_{\infty }(x ; \theta , \beta , m^{\epsilon }=0) \, \hbox {d}x =\frac{1}{\beta \theta }. \end{aligned}$$

#### Proposition 3.1

Consider equations (2.24), (2.26) and (3.9), (3.10). Assume the potential $V^\epsilon$ is smooth and has fluctuations which are truncated in an interval $[-a,a]$. Then the limits $\epsilon \rightarrow 0, \, N\rightarrow \infty , \, T\rightarrow \infty$ (Eqs. (2.24) and (2.26)) and $N\rightarrow \infty , \, T\rightarrow \infty , \, \epsilon \rightarrow 0$ ((3.9) and (3.10)) do not commute. In particular, the $\epsilon \rightarrow 0$ limits of the self-consistency equation (3.4) and of the equation for the critical temperature (3.5) are **different** from (2.24) and (2.26).

#### Proof

The proof of this result follows from properties of periodic functions (Pavliotis and Stuart 2008, Thm. 2.28). Consider $u\in L^2({\mathbb {R}};C_{per}(\mathcal {Y})), \, \epsilon > 0$ and define $u^\epsilon (x,y) = u\left( x,\frac{x}{\epsilon }\right)$. Then

3.6 $$\begin{aligned} u^\epsilon \rightharpoonup \int _\mathcal {Y}u(x,y) \ \hbox {d}y \quad \text{ weakly } \text{ in } \;\; L^2({\mathbb {R}}). \end{aligned}$$

We will use this fact to identify the limits as $\epsilon \rightarrow 0$ of $p^\epsilon _\infty (x;\theta ,\beta ,m^\epsilon )$ and $Z^\epsilon (m^\epsilon ;\theta ,\beta )$, in order to obtain the limits of the first and second moments. First, we note that both the invariant density $p^\epsilon$ and the first moment $m^\epsilon$ depend on $\epsilon$. For a fixed $\epsilon >0$, it is straightforward to check that the two-scale potentials verify the conditions presented in Arnold et al. (1996, Eqs. (3.1), (3.2)), as long as the nonseparable fluctuations are truncated outside the interval $[-a, a]$—this is the case for us; see Table 1 in Sect. 4. This, by estimates (Arnold et al. 1996, Eqs. (3.7), (3.8)), implies uniform boundedness of the first moment, $m^\epsilon$, as well as existence of a unique global weak solution for the McKean–Vlasov equation. We can therefore extract a converging subsequence that converges to some $m \in {\mathbb {R}}$. We use the notation $V_{eff}(x;m,\theta ) = V_0(x) + \theta \left( \frac{1}{2} x^2 - m x\right)$ with $V(x,y) = V_0(x) +V_1(x,y)$—see Eq. (2.7). We note that $V_{eff}$ depends smoothly on *m*. We use the convergence of $m^{\epsilon }$ to *m* and (3.6) to deduce:

3.7 $$\begin{aligned} Z^\epsilon (m^{\epsilon };\theta ,\beta )= & {} \int _{\mathbb {R}}e^{-\beta \left( V_{eff}(x;m^{\epsilon },\theta )+V_1 \left( x, \frac{x}{\epsilon } \right) \right) }\ \hbox {d}x \nonumber \\\rightarrow & {} \int _0^L\int _{\mathbb {R}}e^{-\beta \left( V_{eff}(x;m,\theta ) + V_1(x,y)\right) } \ \hbox {d}x \ \hbox {d}y =: \bar{Z}(m;\theta ,\beta ). \end{aligned}$$

Similarly,

3.8 $$\begin{aligned} \int _{\mathbb {R}}x \ e^{-\beta \left( V_{eff}(x;m^{\epsilon },\theta ) + V_1\left( x,\frac{x}{\epsilon }\right) \right) }\ \hbox {d}x \rightarrow \int _0^L\int _{\mathbb {R}}x \ e^{-\beta \left( V_{eff}(x;m,\theta ) + V_1(x,y)\right) } \ \hbox {d}x \ \hbox {d}y. \end{aligned}$$

Combining (3.7) and (3.8), we obtain

3.9 $$\begin{aligned} m = \frac{1}{\int _0^L\int _{\mathbb {R}}e^{-\beta \left( V_{eff}(x;m,\theta ) + V_1(x,y)\right) } \ \hbox {d}x \ \hbox {d}y} \int _0^L\int _{\mathbb {R}}x \ e^{-\beta \left( V_{eff}(x;m,\theta ) + V_1(x,y)\right) } \ \hbox {d}x \ \hbox {d}y. \end{aligned}$$

Arguing in a similar way for the variance, we conclude that

3.10 $$\begin{aligned} 1 = \frac{\beta \theta }{\bar{Z}(m;\theta ,\beta )} \int _0^L\int _{\mathbb {R}}x^2 \ e^{-\beta \left( V_{eff}(x;m,\theta ) + V_1(x,y)\right) } \ \hbox {d}x \ \hbox {d}y. \end{aligned}$$

We conclude that Eqs. (3.9) and (3.10) are **different**, from (2.24) and (2.26). $\square$

The two limits $\epsilon \rightarrow 0, \, N\rightarrow \infty , \, T\rightarrow \infty$ and $N\rightarrow \infty , \, T\rightarrow \infty , \, \epsilon \rightarrow 0$ commute in the case where the fluctuations in the potential are independent of the macroscale *x*, $V_1 =V_1(y)$ in (2.7). An immediate corollary of the above proposition is the following.

#### Corollary 3.2

Separable fluctuations do not affect the bifurcation diagram in the mean field limit.

#### Proof

When the fluctuations are separable (i.e., $V_1(x,y)$ does not depend on *x*), $\psi (x,\beta )$ in (2.24), (2.26) becomes a constant that we can ignore since it also appears in the partition function and they cancel out. Similarly, the terms of the form $\int _{\mathbb {R}}e^{-\beta V_1(x,y)} \ \hbox {d}y$ in Eqs. (3.9) and (3.10) become constants independent of *x* and cancel with the corresponding terms in the partition function (3.7). $\square$

**Fig. 3:**
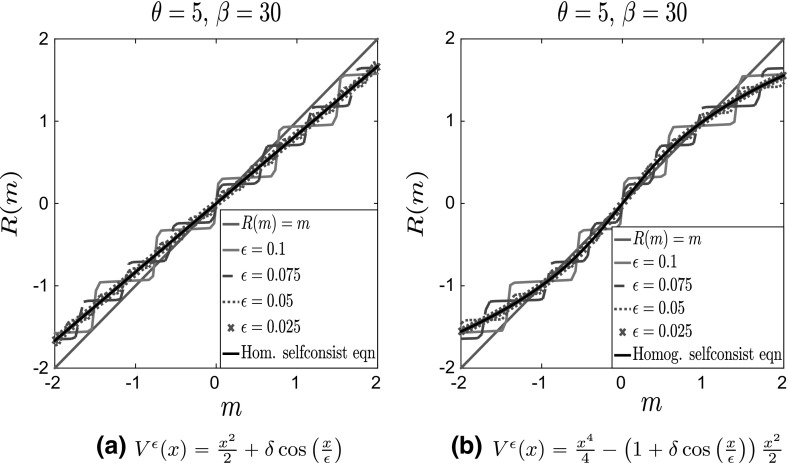
Plot of $R(m^\epsilon ;\theta ,\beta )$ for $\theta = 5, \, \beta = 30, \delta = 1$ and various values of $\epsilon$ for separable potentials. **a** Convex potential $V_0(x)$ and **b** bistable potential $V_0(x)$. For comparison, we also plot the line $y=x$ in solid blue and the solution of the homogenized self-consistency equation in a solid black line (Color figure online)

**Fig. 4:**
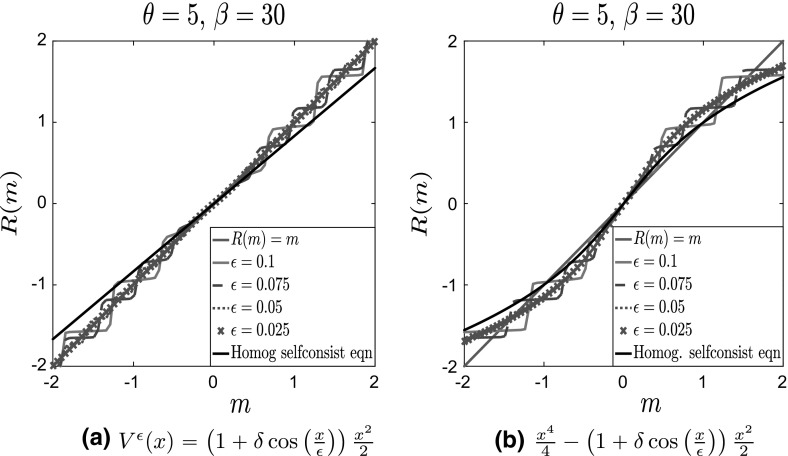
Plot of $R(m^\epsilon ;\theta ,\beta )$ for $\theta = 5, \, \beta = 30, \delta = 1$ and various values of $\epsilon$ for nonseparable potentials. **a** Convex potential $V_0(x)$ and **b** bistable potential $V_0(x)$. For comparison, we also plot the line $y=x$ in solid blue and the solution of the homogenized self-consistency equation in a solid black line (Color figure online)

To illustrate the fact that the two limits do commute when the fluctuations are independent of the macroscale, we present in Figs. 3 and 4 the plots of $R(m^\epsilon ;\theta ,\beta )$ for various values of $\epsilon$ and fixed $\beta$ and $\theta$, which we compare with the solution of the homogenized self-consistency equation $R(m;\theta ,\beta )=m$. We present results both for a convex and nonconvex confining potential, with periodic fluctuations. More details about the two-scale potentials that we use for the numerical simulations will be given in Sect. 4.

As is evident from Fig. 2a, the oscillatory part of the potential introduces (infinitely many) additional local minima. Consequently, Tugaut (2014), the self-consistency equation $R(m^\epsilon ;\theta ,\beta )=m^\epsilon$ has multiple solutions. Furthermore, as shown in Fig. 3b, in the limit $\epsilon \rightarrow 0$, the curves $R(m^\epsilon ;\theta ,\beta )$ (various dashed lines) approach those given by $R(m;\theta ,\beta )$ computed from Eq. (2.24) (full black line), in accordance with Corollary 3.2, showing the commutativity of the two limits.

Let us consider now the case of nonseparable fluctuations. As we have already discussed, see Fig. 2b and also the inside panels of Figs. 7a and 9a, the resulting two-scale potential does not only contain many additional local minima, but it is also flattened around $x=0$. In Fig. 4, we present curves $R(m^\epsilon ;\theta ,\beta )$ for nonseparable fluctuations, compared with the line $R(m;\theta , \beta ) = m$ (or $y=x$). We observe that in the limit $\epsilon \rightarrow 0$ the curves $R(m^\epsilon ;\theta ,\beta )$ (various dashed lines) **do not** converge to $R(m;\theta ,\beta )$ corresponding to the homogenized problem (full black line), in accordance with Prop. 3.1. Notice also the flatness of $R(m^\epsilon ;\theta ,\beta )$ around $m=0$ for smaller values of $\epsilon$, which follows from the flatness of the corresponding potentials $V^\epsilon$ around $x=0$.

### Multiscale Analysis for the McKean–Vlasov Equation in a Two-Scale Confining Potential

In this section, we study the problem of periodic homogenization for the McKean–Vlasov equation in a locally periodic confining potential, for the Curie–Weiss quadratic interaction and in one dimension. We only present formal arguments. The rigorous analysis of this problem will be presented elsewhere.

We consider the nonlinear Fokker–Planck equation (3.2) with $F(x) = \frac{x^2}{2}$:

3.11 $$\begin{aligned} \frac{\partial p^{\epsilon }}{\partial t} = \beta ^{-1}\frac{\partial ^2p^\epsilon }{\partial x^2} + \frac{\partial }{\partial x} \left( V^\prime _0(x)p^{\epsilon } + V_1^\prime \left( x,\frac{x}{\epsilon }\right) p^{\epsilon } + \theta (x-m^\epsilon )p^{\epsilon }\right) , \end{aligned}$$

with initial conditions $p^{\epsilon }(x,0) = p_{in}(x)$, independent of $\epsilon$ and where the prime denotes differentiation with respect to *x*. The PDE (3.11) is coupled to the self-consistency equation

3.12 $$\begin{aligned} m^\epsilon (t) = \int _{\mathbb {R}}x \ p^{\epsilon }(x,t)\ \hbox {d}x. \end{aligned}$$

This homogenization problem is (slightly) different from the standard one for the Fokker–Planck equation in a two-scale potential that was studied in Duncan et al. (2016a), Duncan et al. (2016b) due to the self-consistency equation (3.12). In particular, in addition to the standard two-scale expansion for the solution of the Fokker–Planck equation (3.11), we also need to expand the solution of (3.12) into a power series in $\epsilon$:

3.13a $$\begin{aligned} p^{\epsilon }(x,t)= & {} p_0 \left( x, \frac{x}{\epsilon },t \right) + \epsilon p_1\left( x, \frac{x}{\epsilon },t \right) + \epsilon ^2 p_2\left( x, \frac{x}{\epsilon },t \right) + \dots , \end{aligned}$$

3.13b $$\begin{aligned} m^\epsilon= & {} m_0 + \epsilon m_1 + \epsilon ^2m_2+ \dots , \end{aligned}$$

where, as usual (Pavliotis and Stuart 2008), we take $\{ p_j = p_j\left( x ,\cdot , t \right) , \; j=0, 1, \dots \}$ to be *L*-periodic in their second argument. Substituting (3.13) into (3.11) and (3.12) and using the standard tools from the theory of periodic homogenization, e.g., Fredholm’s alternative, we obtain the homogenized equation (2.19), satisfied by the marginal of the first term in the two-scale expansion $p(x,t) = \int _0^L p(x,y,t) \, \hbox {d}y$ and with the partial free energy $\psi (x)$ given by (2.20) and with

$$\begin{aligned} m(t):=m_0(t) = \int _{{\mathbb {R}}} \int _0^L x p_0(x,y,t) \, \hbox {d}y \hbox {d}x. \end{aligned}$$

The convergence of $m^{\epsilon }(t)$ to *m*(*t*) can be justified using the a priori estimates on moments of the solution to the McKean–Vlasov equation that were derived in Arnold et al. (1996), in particular (Arnold et al. 1996, Eqs. (3.1), (3.2)).

Alternatively, we can work with the backward Kolmogorov equation: We recall that Eq. (3.11) corresponds to the McKean SDE

3.14 $$\begin{aligned} \hbox {d}\mathbf{x}_t =-\left[ {V^{\epsilon }}^{\prime } \left( \mathbf{x}_t\right) + \theta (\mathbf{x}_t-m^\epsilon )\right] \ \hbox {d}t + \sqrt{2\beta ^{-1}} \hbox {d}B_t, \end{aligned}$$

with $V^{\epsilon }(x) = V \left( x, \frac{x}{\epsilon } \right)$. We introduce the auxiliary variable $y_t = \frac{\mathbf{x}_t}{\epsilon }$, see, e.g., Pavliotis and Stuart (2007), and using the chain rule, we can write (3.14) as a system of interacting diffusions across scales, driven by the same Brownian motion,

3.15 $$\begin{aligned} \hbox {d}\mathbf{x}_t&= -\left[ \partial _xV\left( \mathbf{x}_t,y_t\right) +\frac{1}{\epsilon }\partial _y V\left( \mathbf{x}_t,y_t\right) + \theta (\mathbf{x}_t-m^\epsilon )\right] \ \hbox {d}t + \sqrt{2\beta ^{-1}} \hbox {d}B_t,\end{aligned}$$

3.16 $$\begin{aligned} \hbox {d}y_t&= -\left[ \frac{1}{\epsilon }\partial _xV \left( \mathbf{x}_t,y_t\right) +\frac{1}{\epsilon ^2}\partial _yV \left( \mathbf{x}_t,y_t\right) + \frac{\theta }{\epsilon }(\mathbf{x}_t-m^\epsilon )\right] \ \hbox {d}t + \sqrt{\frac{2\beta ^{-1}}{\epsilon ^2}} \hbox {d}B_t. \end{aligned}$$

We start by expanding the first moment $m^\epsilon$ in powers of $\epsilon$ as in (3.13b). The backward Kolmogorov equation for the observable $u^{\epsilon }(x,y,t) = {\mathbb {E}}(f(x^{\epsilon }_t, y^{\epsilon }_t) | x^{\epsilon }_0 = x, y^{\epsilon }_0 = y)$ reads (neglecting terms of $O(\epsilon )$ that are due to the expansion of $m^\epsilon$)

3.17a $$\begin{aligned} \frac{\partial u^{\epsilon }}{\partial t}= & {} \left( \frac{1}{\epsilon ^2}{\mathcal {L}}_0+\frac{1}{\epsilon }{\mathcal {L}}_1+{\mathcal {L}}_2\right) u^{\epsilon }, \end{aligned}$$

3.17b $$\begin{aligned} u^{\epsilon }(x,y,0)= & {} f(x,y), \end{aligned}$$

with

$$\begin{aligned} \mathcal {L}_0= & {} - \partial _y V \partial _y - \beta ^{-1} \partial _y^2, \\ \mathcal {L}_1= & {} -\left( \partial _x V -\theta (x - m_0) \right) \partial _y - \partial _y V \partial _x - 2 \beta ^{-1} \partial _x \partial _y, \\ \mathcal {L}_2= & {} - \left( \partial _x V -\theta (x - m_0) \right) \partial _x - \theta m_1 \partial _y - \beta ^{-1} \partial _x^2, \end{aligned}$$

We can now proceed with the analysis of (3.17a), first for the choice $f(x) = x$, i.e., the evolution of the first moment, and then for arbitrary observables. We obtain, thus, the homogenized backward Kolmogorov equation, from which we can read off the homogenized McKean SDE and the corresponding Fokker–Planck equation:

3.18 $$\begin{aligned} \frac{\partial p}{\partial t}= & {} \frac{\partial }{\partial x}\left[ \beta ^{-1}\frac{\partial \left( {\mathcal {M}}(x) p\right) }{\partial x} + {\mathcal {M}}(x)\Big ( V'_0(x) +\psi '(x) + \theta \left( x - m(t)\right) \Big )p\right. \nonumber \\&\left. +\, \beta ^{-1}\frac{\partial {\mathcal {M}}(x)}{\partial x}p\right] , \end{aligned}$$

where $\psi (x) = -\beta ^{-1}\ln \left( \int _0^L e^{-\beta V_1(x,y)} \ \hbox {d}y \right)$ and ${\mathcal {M}}(x)$ is defined in (2.10). For the sake of brevity, we will omit the details.

## Numerical Simulations

In this section, we construct the bifurcation diagram for the stationary McKean–Vlasov equation (both for finite values of $\epsilon$ and in the homogenization limit), present the results of Monte Carlo (MC) simulations based on the numerical solution of the particle/SDE approximation and also solve the time-dependent McKean–Vlasov PDE. Our goal is to investigate numerically the issue of (lack of) commutativity of the mean field and homogenization limits. We consider interacting diffusions (and the corresponding McKean–Vlasov) in one dimension and we study two types of large-scale and fluctuating parts of the potential. We consider both convex and nonconvex potentials, and both additive (separable) and multiplicative (nonseprarable) fluctuations. The four potentials that we use for our simulations are tabulated in Table 1. We remark that the nonseparable fluctuations $V_1^\times (x)$ are truncated outside the interval $[-a,a]$ in order to prevent the oscillations from growing as $|x| \rightarrow +\infty$.^6^ We note that this is necessary for the proof of the homogenization theorem in Duncan et al. (2016b) and that, furthermore, it ensures that the a priori estimates on the moments from Arnold et al. (1996) hold.^7^

**Table 1:** Potentials used for the numerical simulations

Confining potential $V_0(x)$	Fluctuating potential $V_1(x)$	Case
$V_0^c(x) = \frac{x^2}{2}$	$V_1^+(x) = \delta \cos \left( \frac{x}{\epsilon }\right)$	1
	$V_1^\times (x) = \delta \chi _{[-a,a]}(x)\frac{x^2}{2}\cos \left( \frac{x}{\epsilon }\right)$	2
$V_0^b(x) = \frac{x^4}{4} - \frac{x^2}{2}$	$V_1^+(x) = \delta \cos \left( \frac{x}{\epsilon }\right)$	3
	$V_1^\times (x) = \delta \chi _{[-a,a]}(x)\frac{x^2}{2}\cos \left( \frac{x}{\epsilon }\right)$	4

Throughout this section, we consider fluctuations which have period $L=2\pi$. In all cases, we will consider the Curie–Weiss interaction potential $F(x) = \frac{x^2}{2}$, and throughout Sections 4.1 and 4.2, we will fix the interaction strength to be $\theta = 5$. We choose this value because larger values of $\theta$ allow for bifurcations to occur at higher temperatures, i.e., lower $\beta$, which is easier to handle numerically. In fact, the relevant bifurcation parameter for our problem is given by the combination $\beta \theta$; see Eq. (1.10). Fixing $\theta$ allows us to construct the bifurcation diagram by varying only the temperature. It is also clear from Eq. (1.10) that this equation has no solutions for negative values of $\theta$, i.e., that no (pitchfork) bifurcations can occur for $\theta < 0$.

Using Eq. (2.16), we note that the diffusion coefficient for separable fluctuations in the potential is independent of *x* and is given by

4.1 $$\begin{aligned} {\mathcal {M}}^{+}(x) = \frac{1}{\left( \frac{1}{ 2 \pi }\int _0^{2\pi } e^{-\beta V_1^{+}(x,y)} \ \hbox {d}y\right) \left( \frac{1}{ 2 \pi }\int _0^{2\pi } e^{\beta V_1^{+}(x,z)} \ dz\right) } = \frac{1}{I_0(\beta )I_0(-\beta )}, \end{aligned}$$

where $I_0(\cdot )$ is the modified Bessel function of the first kind (Duncan et al. 2016a). On the other hand, for nonseparable fluctuations (cases 2 and 4 in Table 1) we obtain

4.2 $$\begin{aligned} {\mathcal {M}}^{\times }(x) = \frac{1}{\left( \frac{1}{ 2 \pi }\int _0^{2\pi } e^{-\beta V_1^{\times }(x,y)} \ \hbox {d}y\right) \left( \frac{1}{ 2 \pi }\int _0^{2\pi } e^{\beta V_1^{\times }(x,z)} \ dz\right) } = \frac{1}{I_0\left( \beta \frac{x^2}{2}\right) I_0\left( -\beta \frac{x^2}{2}\right) }. \end{aligned}$$

Furthermore, we obtain the following formulas for the partition functions

4.3 $$\begin{aligned} {\mathcal {Z}}^{+}(x) = e^{-\beta \left( V_0(x)+\theta \left( \frac{x^2}{2}-m x\right) \right) }I_0(\beta ), \quad {\mathcal {Z}}^{\times }(x) = e^{-\beta \left( V_0(x)+\theta \left( \frac{x^2}{2}-m x\right) \right) }I_0\left( \beta \frac{x^2}{2}\right) . \end{aligned}$$

We can now solve the self-consistency equation (1.9) and the equation for the critical temperature (1.10) for the various potentials given in Table 1. We will track each branch of the bifurcation diagram using arclength continuation, which will enable us to plot the first moment *m* as a function of the inverse temperature $\beta$ for a fixed value of the interaction strength $\theta$. We do this using the Moore–Penrose quasi-arclength continuation algorithm.^8^ The stability of each branch was determined in two different ways: First, we checked whether it corresponded to a local minimum or maximum of the confining potential. Second, we solved the time-dependent McKean–Vlasov equation—see details in Sect. 4.5—using a perturbation of the steady state belonging to each branch (for a particular value of $\beta$ and $\theta$) as initial condition. Finally, we have confirmed the stability of each branch by computing the free energy (1.5) of a steady state from that branch at a particular value of $\beta$, chosen so that all the branches plotted were present. Stable branches, plotted in blue in all the figures presented in this section, correspond to local minimizers of the free energy functional; unstable branches, plotted in red, correspond to local maxima of the free energy.

### Mean Field Limit of the Homogenized System of SDEs: The $\epsilon \rightarrow 0, \, N\rightarrow \infty$ Limit

**Fig. 5:**
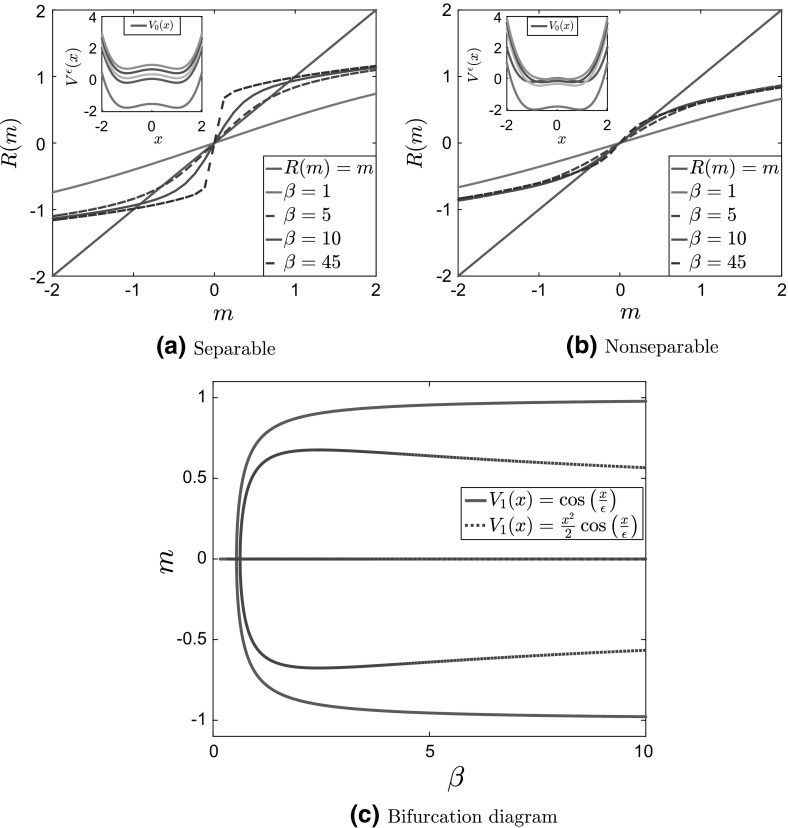
Plot of $R(m;\theta ,\beta )$ compared to the diagonal $y=x$ ( $R(m;\theta ,\beta )=m$) for $\theta = 5, \delta = 1, \, a = 5$ and various values of $\beta$ for the homogenized bistable potentials with **a** separable fluctuations (potentials for various values of $\beta$ shown on the inside panel), and **b** nonseparable fluctuations (potentials for various values of $\beta$ shown on the inside panel). **c** Bifurcation diagram of *m* as a function of $\beta$ for the potentials in (5a) (full line) and (5b) (dashed line)

As discussed before (see discussion of Corollary 3.2), when the fluctuations are separable the partial free energy $\psi (x)$ defined in Eq. (2.20) drops out from the homogenized stationary Fokker–Planck equation. This implies, in particular, that the invariant measure(s) of the homogenized dynamics is(are) independent of the fluctuating part of the potential. In particular, there are still no phase transitions when the large-scale part of the potential is convex and still only one pitchfork bifurcation for the bistable potential case—see Fig. 5—where two new, stable, branches emerge from the zero mean solution. We note that in this case the homogenized confining potential in the homogenized equation depends on the inverse temperature $\beta$; see the inside panels in Fig. 5. In particular, the values of the local minima of the effective potential are shifted, although their location remains the same, and there are no changes in the topology of the bifurcation diagrams.

For nonseparable fluctuations, the mean field and homogenization limits do not commute (see Prop. 3.1). In fact, the homogenization procedure can convexify the effective potential, and we still see no bifurcations when the large-scale part of the potential is convex, while for the bistable potential there is still only one phase transition. The effect of fluctuations on the bifurcation diagram is visible by a shift of the critical temperature at which the phase transition occurs.

Since there are no phase transitions for the convex potential (cases 1 and 2 in Table 1), we do not present numerical results for this case. We present in Fig. 5 the plots of $R(m;\theta ,\beta )$ and the bifurcation diagrams for the bistable potential with separable and nonseparable fluctuations (cases 3 and 4, respectively). We observe that, for nonseparable fluctuations, the function $R(m;\theta ,\beta )$ is flat around $m=0$; see Fig. 5b. As we have already mentioned, the topology of the bifurcation diagram does not change, in comparison with that of the bistable potential $V_0^b(x)$ with no fluctuations (see Fig. 1b for this case); thus, the effect of fluctuations is only observed by a shift in the critical temperature.

### Mean Field Limit of the Multiscale System of SDEs: Effects of Finite $\epsilon$

In this section, we present numerical results on the bifurcation diagram when we first pass to the mean field limit, while keeping $\epsilon$ small but finite. We are particularly interested in the finite $\epsilon$ effects on the bifurcation diagrams for the two-scale potentials presented in Table 1.

#### Convex Confining Potential with Separable and Nonseparable Fluctuations

**Fig. 6:**
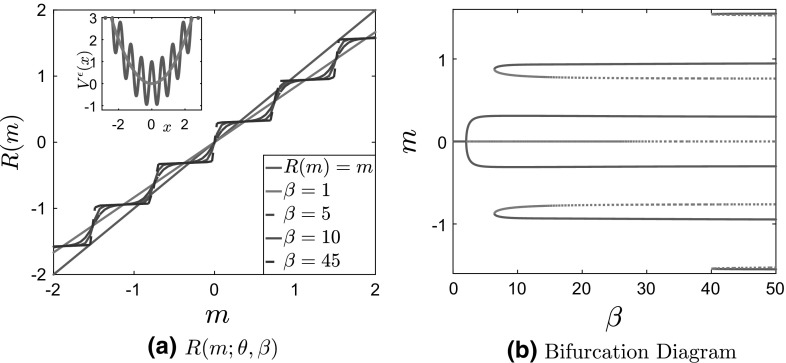
Results for case 1: convex $$V_0^c$$ with separable fluctuations, for $$\theta = 5, \, \delta = 1, \, \epsilon = 0.1$$. **a** $$R(m^\epsilon ;\theta ,\beta )$$ for various values of $$\beta $$, with the potential $$V^\epsilon (x)$$ (full line) compared with $$V_0^c(x) $$ (dashed line) in the inside panel. **b** Bifurcation diagram of *m* as a function of $$\beta $$. Full lines correspond to stable solutions, while dashed lines represent unstable ones

We first consider Case 1 in Table 1: a convex large-scale potential with separable fluctuations. We present in Fig. 6 the solution to the self-consistency equation $R(m;\theta ,\beta )=m$, the two-scale potential, and the bifurcation diagram for this case. For all finite values of $\epsilon$, the resulting potential is nonconvex. This results in the self-consistency equation having multiple solutions (in fact, as $\epsilon \rightarrow 0$, there are infinitely many solutions). In addition to the emerging pitchfork bifurcation (second-order, or continuous, phase transition), we observe the emergence of discontinuous branches that correspond to metastable states, since they are not (global) minimizers of the free energy; see the results presented in Table 2.

**Table 2:** Free energy of a steady state in each branch of Figs. 6, 7, 8 and 9 for fixed values of $\beta$

Figure	6	7	8	9
$\beta$	45	29	20	8
Free Energy	$\ 0.3080$	0.1441	$-\,0.5827$	$-\,1.7409$
	0.3066	0.3684	$-\,0.5674$	$-\,0.9933$
	$-\,0.4600$	0.1433	$-\,1.0918$	$-\,0.8241$
	$-\,0.3908$	0.3184	$-\,0.7727$	0.0856
	$-\,0.8593$	0.0976	$-\,0.8868$	
	$-\,0.6514$	0.2425	$-\,0.6903$	
		0.0625		
		0.0630		
		0.0586		

**Fig. 7:**
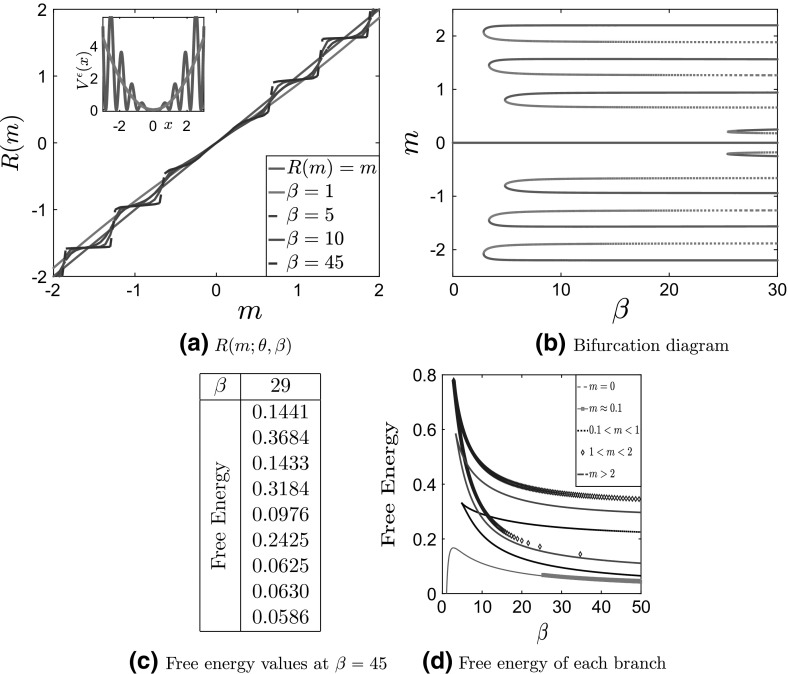
Results for case 2: convex $$V_0$$ with nonseparable fluctuations, for $$\theta = 5, \, \delta = 1, \, \epsilon = 0.1$$. **a** $$R(m;\theta ,\beta )$$ for various values of $$\beta $$, with the potential $$V^\epsilon (x)$$ (full line) compared with $$V_0^c(x) $$ (dashed line) in the inside panel. **b** Bifurcation diagram of *m* as a function of $$\beta $$. Full lines correspond to stable solutions, while dashed lines represent unstable ones. **c** Values of the free energy of the steady state in each branch of (**b**) for $$\beta = 45$$. **d** Free energy of each branch of the bifurcation diagram

Next, we consider the second case in Table 1: a convex large-scale potential $V_0^c(x)$ with nonseparable fluctuations. Similarly, we present in Fig. 7 the solution to the self-consistency equation $R(m^\epsilon ;\theta ,\beta )=m^\epsilon$, the two-scale potential and the bifurcation diagram. We note that, as we mentioned before, we restrict the nonseparable fluctuations to a finite interval. In our computations, we use $a=5$, in the characteristic function in Table 1.

We observe in Fig. 7b that no pitchfork bifurcations appear; all new branches that appear do not emerge continuously from the mean-zero solution. This is due to the flatness observed in the potential around $m=0$ (see Fig. 7a). Furthermore, the mean-zero solution remains the global minimizer of the free energy for all values of $\beta$. This is tabulated in Table 7c, where they are listed in the same way as in Table 2, i.e., in decreasing order of nonnegative *m*. The free energies of the different branches are presented in Fig. 7d. These new branches correspond to metastable states.

We have checked the stability of each branch by computing the free energy (1.5) of a steady state from that branch at a particular value of $\beta$, chosen so that all the branches plotted were present. We summarize the results in Table 2. Since we only consider symmetric potentials, it is sufficient to calculate the free energy for the branches with, say, nonnegative values of *m*. In each column of Table 2, the values of the free energy are presented from the branch with largest value of *m* to the lowest; the last value presented in each column corresponds to the branch with $m=0$. We summarize the results in Table 2.

We observe that the branch corresponding to a pitchfork bifurcation (i.e., second-order phase transition), when present, has the lowest value of the free energy, i.e., it is the globally stable one. Furthermore, when a pitchfork bifurcation does not occur—see Fig. 7—the branch corresponding to $m=0$ is the one with the lowest value of the free energy. Finally, we observe that the stability of the branches in Fig. 9b does not alternate in the same manner as in the previous figures. This is due to the flatteness of the potential around $x=0$ for nonseparable oscillations.

The results on the stability of the different branches that are reported in this section are preliminary. A more thorough study of the local (linear) and global stability of the stationary states of the McKean–Vlasov dynamics in multiwell potentials will be presented elsewhere. We mention in passing the early rigorous work on the global stability of the steady states for the McKean–Vlasov equation in Tamura (1987) as well as the careful study of the connection between the loss of linear stability of the uniform state and phase transitions for the McKean–Vlasov equation on the torus (without a confining potential) and with finite-range interactions in Chayes and Panferov (2010).

#### Bistable Confining Potential with Separable and Nonseparable Fluctuations

Here we consider cases 3 and 4 in Table 1, the bistable potential $V_0^b(x)$. In this case, the large-scale potential exhibits a second-order phase transition even in the absence of small-scale fluctuations (see the pitchfork bifurcation in Fig. 1b) due to the existence of two local minima for $V_0^b(x)$. We are interested in analyzing the topological changes that rapid oscillations in the potential induce to the bifurcation diagram.

**Fig. 8:**
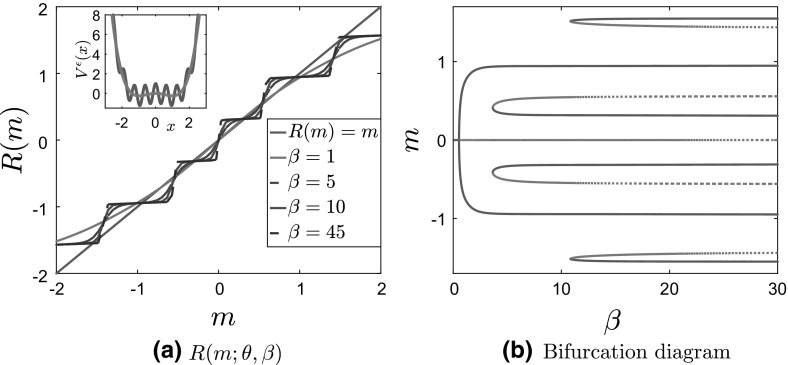
Results for case 3: bistable $$V_0^b$$ with separable fluctuations, for $$\theta = 5, \, \delta = 1, \, \epsilon = 0.1$$. **a** $$R(m^\epsilon ;\theta ,\beta )$$ for various values of $$\beta $$, with the potential $$V^\epsilon (x)$$ (full line) compared with $$V_0^b(x) $$ (dashed line) in the inside panel. **b** Bifurcation diagram of *m* as a function of $$\beta $$. Full lines correspond to stable solutions, while dashed lines represent unstable ones

We start with separable potentials—see Fig. 8. We observe that the self-consistency equation $R(m^\epsilon ;\theta ,\beta )=m^\epsilon$ exhibits a larger number of solutions for finite $\epsilon$, which, as for the convex case, result in the emergence of metastable states that are not continuously connected with the mean-zero Gibbs state.

Similarly, for the last case in Table 1, case 4 (bistable potential $V_0^b(x)$ and nonseparable fluctuations), there are more solutions to the self-consistency equation. However, the flatness of the potential (and therefore of the curves $R(m;\theta ,\beta )$ near $m=0$) reduces the number of additional branches. Moreover, the topological structure of the bifurcation diagram changes, and we now observe a nonparabolic curve for the main branch, which bifurcates from the mean-zero solution via a pitchfork bifurcation.

**Fig. 9:**
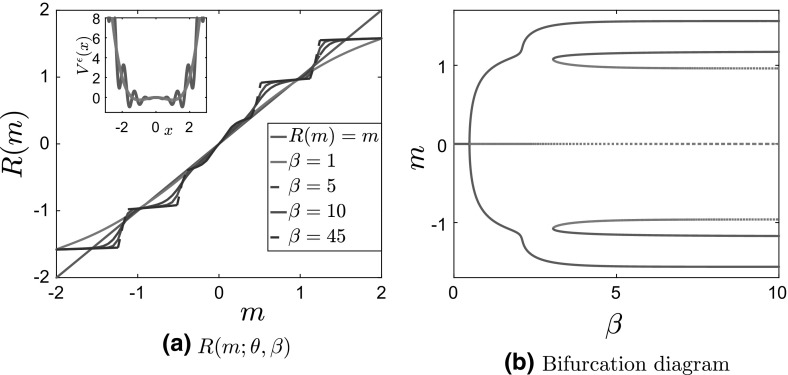
Results for case 4: bistable $$V_0^b$$ with nonseparable fluctuations, for $$\theta = 5, \, \delta = 1, \, \epsilon = 0.1$$. **a** $$R(m^\epsilon ;\theta ,\beta )$$ for various values of $$\beta $$, with the potential $$V^\epsilon (x)$$ (full line) compared with $$V_0^b(x) $$ (dashed line) in the inside panel. **b** Bifurcation diagram of *m* as a function of $$\beta $$. Full lines correspond to stable solutions, while dashed lines represent unstable ones

### Numerical Study of the Critical Temperature as a Function of $\epsilon$

Here we study the influence of finite $\epsilon$ on the critical temperature $\beta _C$, the solution of (3.10) for two-scale potentials, after which continuous phase transitions (pitchfork bifurcations) occur. We do this by solving the equation (we only consider symmetric potentials)

4.4 $$\begin{aligned} \theta ^{-1}\beta ^{-1} = \int _{\mathbb {R}}x^2 p_\infty (x;\theta ,\beta ,m^\epsilon =0) \ \hbox {d}x, \end{aligned}$$

for the various potentials in Table 1.

**Fig. 10:**
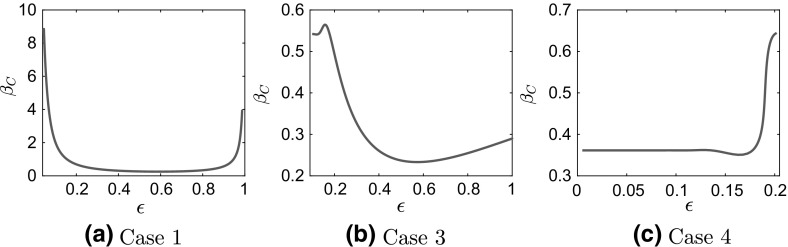
Critical temperature $\beta _C$ as a function of $\epsilon$ for the multiscale Fokker–Planck equation with $\theta = 5$ for cases **a** $1 - V^\epsilon (x) = \frac{x^2}{2} + \delta \cos \left( \frac{x}{\epsilon }\right)$, **b** $3 - V^\epsilon (x) = \frac{x^4}{4} - \frac{x^2}{2} + \delta \cos \left( \frac{x}{\epsilon }\right)$, and **c** $4 - V^\epsilon (x) = \frac{x^4}{4} -\frac{x^2}{2}\left( 1 - \delta \cos \left( \frac{x}{\epsilon }\right) \right)$ in Table 1

We present in Fig. 10 plots of the critical temperature, $\beta _C$ as a function of $\epsilon$ for a fixed $\theta = 5$. The results are presented for cases 1 (Fig. 10a), 3 (Fig. 10b) and 4 (Fig. 10c) from Table 1. We do not present the remaining case because, as shown in Fig. 7b, there is no pitchfork bifurcation from the mean-zero solution for case 2. The dependence of the critical temperature on $\epsilon$ is different for separable and nonseparable potentials. It appears that the critical temperature can change considerable by varying $\epsilon$, which implies that a different number of branches might be present in the bifurcation diagram at a fixed temperature, for different values of $\epsilon$. This issue will be studied in detail in future work.

### Simulations of the Interacting Particles System

In this section, we present the results of Monte Carlo (MC) simulations for the system of interacting diffusions, both for the full, i.e., $\epsilon$-dependent, (2.1) and for the homogenized dynamics (2.17). Our focus is on the study of the convergence of the interacting particles system to their equilibrium state. It should be emphasized that no phase transitions occur for the finite-dimensional particles system. However, the numerical simulation of the two interacting particles systems, (2.1) and the homogenized particle system (2.17) clearly exhibit the lack of commutativity between the mean field and homogenization limits.

For the full dynamics (2.1), we used $\delta = 1$ and $\epsilon = 0.1$. We solved the SDEs using the Euler–Maruyama scheme. For the homogenized dynamics (2.17), since the noise is multiplicative (for nonseparable potentials), we used the Milstein scheme. In both cases, the time step used was $\hbox {d}t = 0.01$, which is of $O(\epsilon ^2)$. Finally, in both cases we initialized the *N* particles as being normally distributed, with mean zero and variance 4, which was large enough so that all the local minima were contained within two standard deviations of the Gaussian distribution.

**Fig. 11:**
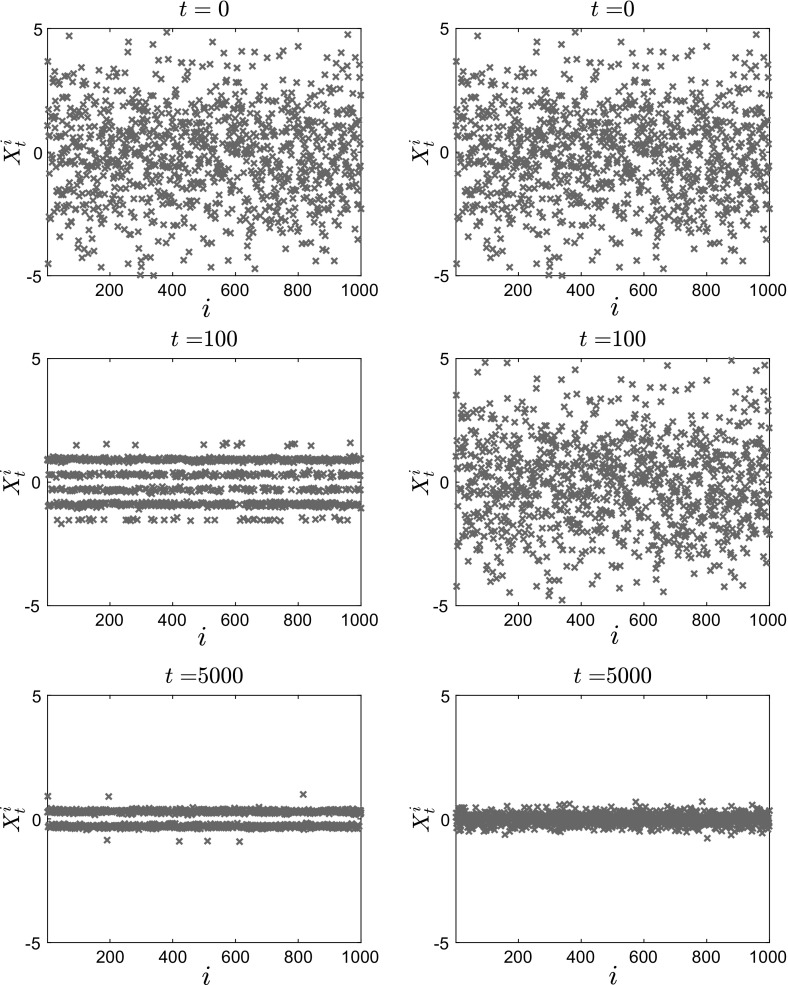
Position of $N=1000$ particles for $V^{\epsilon }(x) = \frac{x^2}{2} + \delta \cos \left( \frac{x}{\epsilon } \right)$, with $\theta = 2$, $\beta = 8$, $\delta = 1$. Left: Eq. (2.1) with $\epsilon = 0.1$. Right: homogenized SDEs (2.17)

**Fig. 12:**
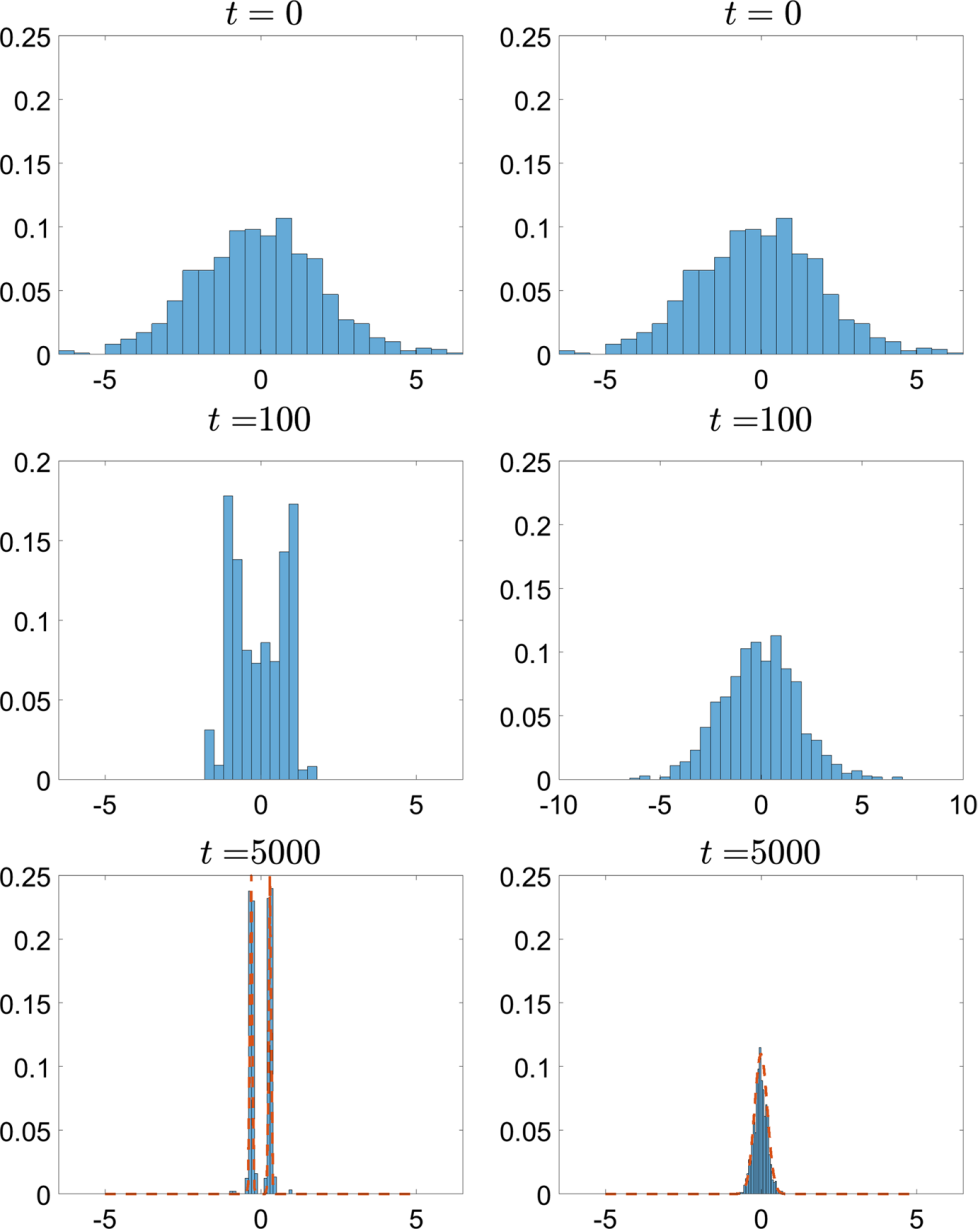
Histogram of $N=1000$ particles for $V^{\epsilon }(x) = \frac{x^2}{2} + \delta \cos \left( \frac{x}{\epsilon } \right)$, with $\theta = 2$, $\beta = 8$, $\delta = 1$. Left: Eq. (2.1) with $\epsilon = 0.1$. Right: homogenized SDEs (2.17)

**Fig. 13:**
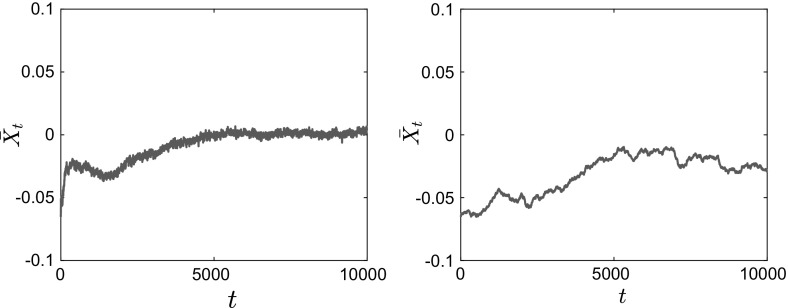
Time evolution of the mean $\bar{X}_t^N = \frac{1}{N}\sum _{i=1}^N X_t^i$ of $N=1000$ particles for $V^{\epsilon }(x) = \frac{x^2}{2} + \delta \cos \left( \frac{x}{\epsilon } \right)$, with $\theta = 2$, $\beta = 8$, $\delta = 1$. Left: Eq. (2.1) with $\epsilon = 0.1$. Right: homogenized SDEs (2.17)

In Figs. 11, 12 and 13, we present the results of our simulations for case 1 in Table 1, the convex potential with separable fluctuations $V^{\epsilon }(x) = \frac{x^2}{2} + \delta \cos \left( \frac{x}{\epsilon } \right)$. In Fig. 11, we present snapshots of the position of each of the $N=1000$ particles for $t=0$ (top panels), $t=100$ (middle panels) and $t=5000$ (bottom panels). The left panels show the results for $\epsilon =0.1$, while the right panels show the results for the homogenized system. In Fig. 12, we present snapshots of the histogram for the $N=1000$ particles for the same time and parameter values, which are $\delta = 1, \, \epsilon = 0.1, \, \theta = 2$ and $\beta = 8$. On the $t=5000$ snapshot, we superpose the corresponding invariant measure, rescaled for comparison, and we observe that the empirical density of the system of interacting diffusions converges to the steady-state solution computed by solving the stationary McKean–Vlasov equation.

We also calculate the empirical average of the interacting particle system

4.5 $$\begin{aligned} \bar{X}_t^N := \frac{1}{N}\sum _{i=1}^N X_t^i, \end{aligned}$$

as a function of time *t*. We observe that in both cases, the average converges to 0 as expected, but that the convergence for the homogenized SDE (2.17) is slower. The position of the *N* particles follows approximately the same qualitative behavior (with the particles clustering close to 0), but as we can see from the corresponding histogram there exist additional wells (nonconvexity) for the finite $\epsilon$ case.

**Fig. 14:**
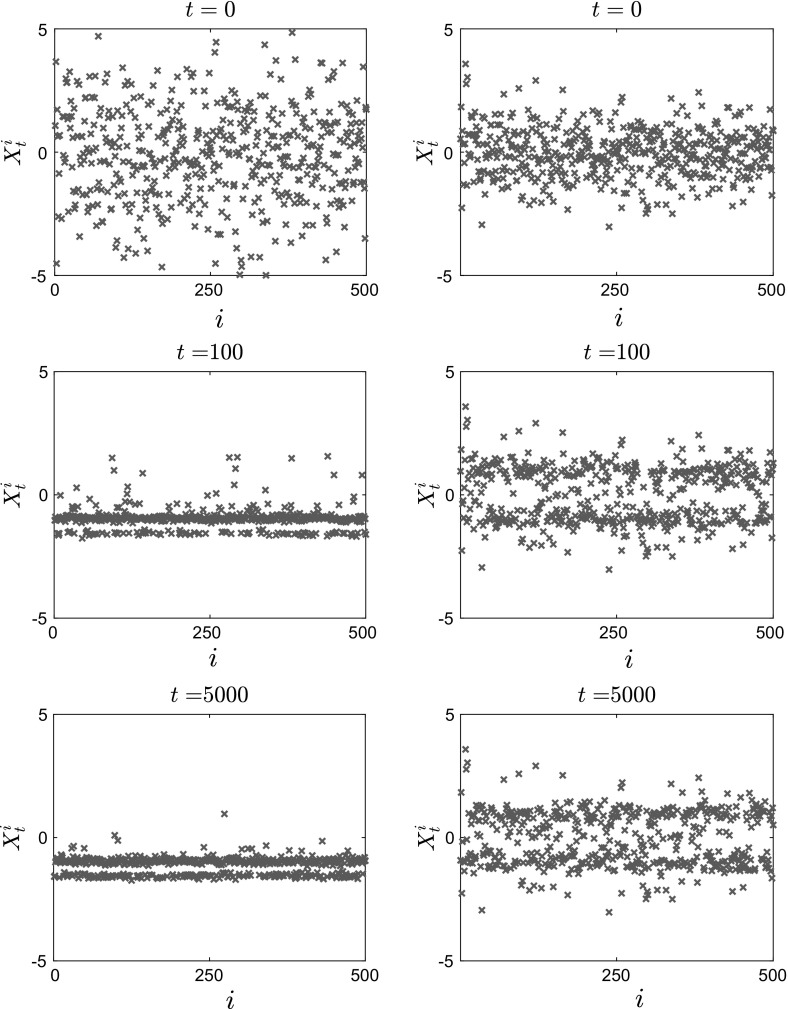
Position of $N=500$ particles for $V^{\epsilon }(x) = \frac{x^4}{4} - \frac{x^2}{2}\left( 1- \delta \cos \left( \frac{x}{\epsilon } \right) \right)$, with $\theta = 0.5$, $\beta \approx 5.6$, $\delta = 1$. Left: Eq. (2.1) with $\epsilon = 0.1$. Right: homogenized SDEs (2.17)

**Fig. 15:**
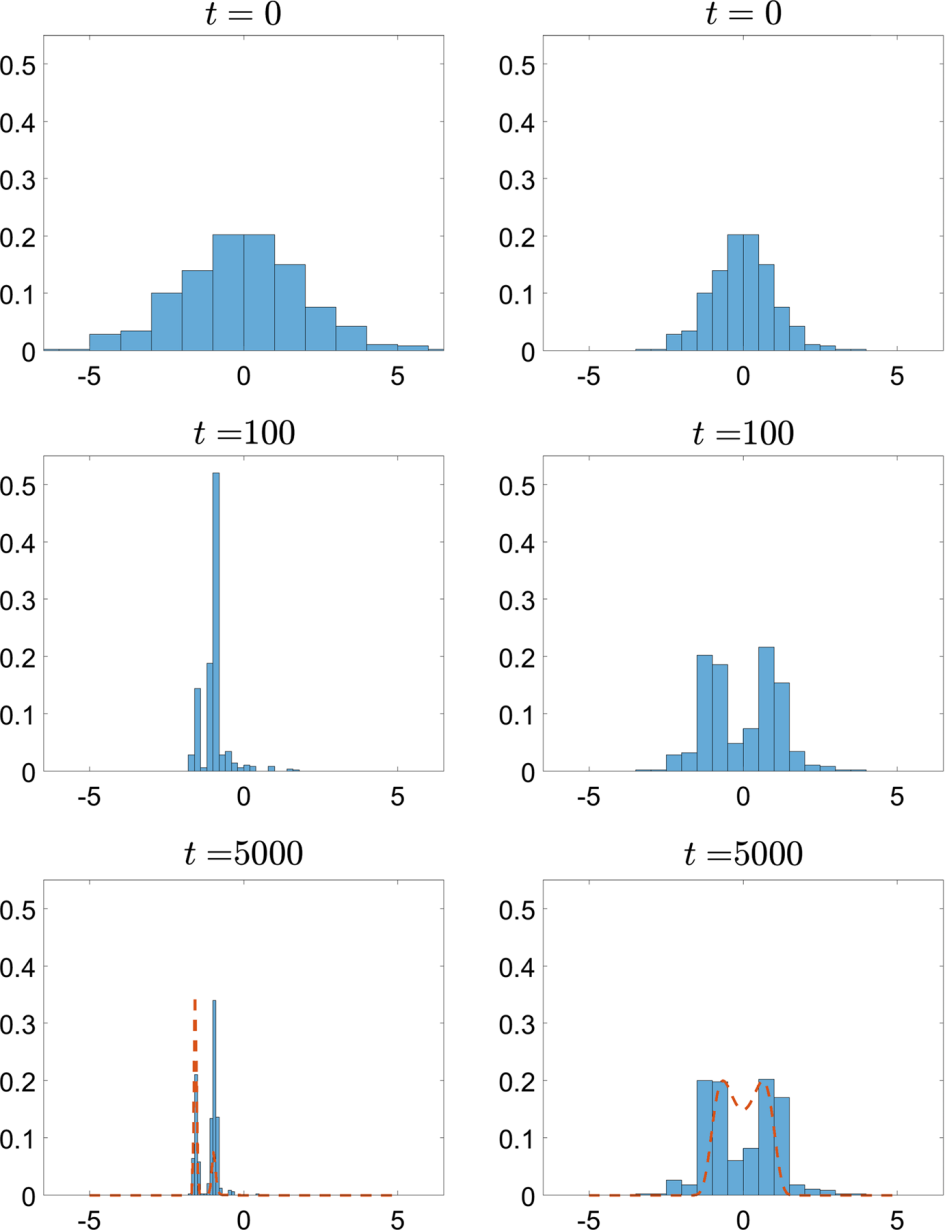
Histogram of $N=500$ particles for $V^{\epsilon }(x) = \frac{x^4}{4} - \frac{x^2}{2}\left( 1- \delta \cos \left( \frac{x}{\epsilon } \right) \right)$, with $\theta = 0.5$, $\beta \approx 5.6$, $\delta = 1$. Left: Eq. (2.1) with $\epsilon = 0.1$. Right: homogenized SDEs (2.17)

**Fig. 16:**
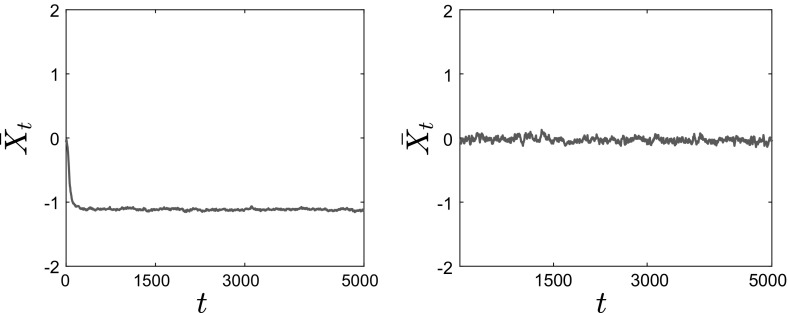
Time evolution of the average $\bar{X}_t^N = \frac{1}{N}\sum _{i=1}^N X_t^i$ of $N=500$ particles for $V^{\epsilon }(x) = \frac{x^4}{4} - \frac{x^2}{2}\left( 1- \delta \cos \left( \frac{x}{\epsilon } \right) \right)$, with $\theta = 0.5$, $\beta \approx 5.6$, $\delta = 1$. Left: Eq. (2.1) with $\epsilon = 0.1$. Right: homogenized SDEs (2.17)

We performed similar experiments for case 4 in Table 1 (i.e., $V^\epsilon (x) = \frac{x^4}{4}-\frac{x^2}{2}\left( 1-\delta \chi _{[-a,a]}(x)\cos \left( \frac{x}{\epsilon }\right) \right)$). Here we used $N=500$ particles, and smaller values of $\theta$ and $\beta$. The parameters used were $\theta = 0.5$, $\beta \approx 5.6$, $\delta = 1$ and $\epsilon = 0.1$, and the results are plotted in Figs. 14, 15 and 16.

In Fig. 14, we present snapshots of the position of each of the $N=500$ particles for $t=0$ (top panels), $t=100$ (middle panels) and $t=5000$ (bottom panels). The left panels show the results for $\epsilon =0.1$, while the right panels show the results for the homogenized SDE (2.17). Here we can observe the noncommutativity of the limits: The particles evolve toward different steady states, which shows the effect of the fluctuations on the critical temperature $\beta _C$ at which phase transitions occur. This will be confirmed below when we present the mean value of the solution.

**Fig. 17:**
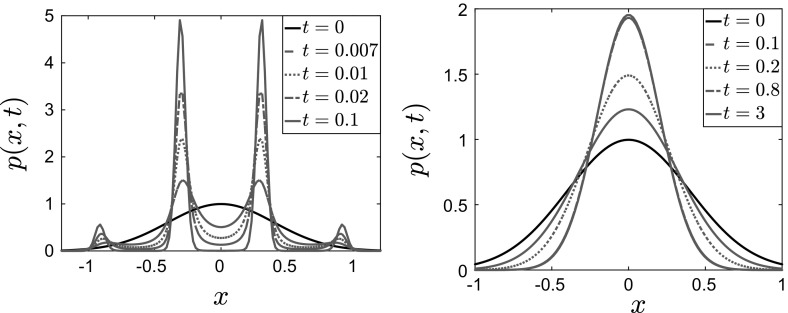
Time evolution of the McKean–Vlasov equation for $V^\epsilon (x) = \frac{x^2}{2} + \delta \cos \left( \frac{x}{\epsilon }\right)$with $\theta = 2$, $\beta =8$, $\delta = 1$. Left: (4.6) with $\epsilon = 0.1$. Right: homogenized equation (4.7)

**Fig. 18:**
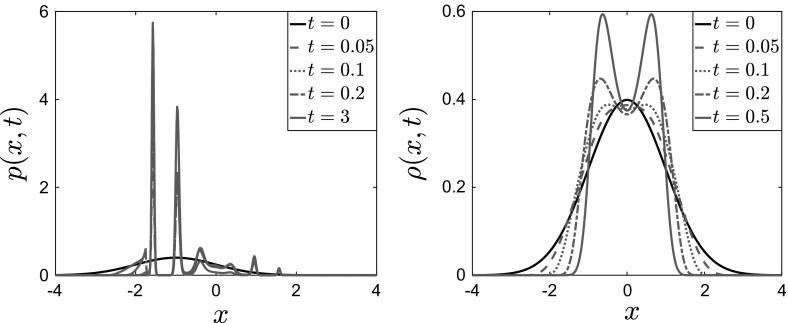
Time evolution of the McKean–Vlasov equation for $V^\epsilon (x) = \frac{x^4}{4} -\frac{x^2}{2}\left( 1- \delta \chi _{[-\,5,5]}(x)\cos \left( \frac{x}{\epsilon }\right) \right)$ with $\theta = 0.5$, $\beta \approx 5.6$, $\delta = 1$. Left: (4.6) with $\epsilon = 0.1$. Right: homogenized equation (4.7)

We present in Fig. 15 snapshots of the histogram of the $N=500$ particles at $t=0, \, t=100$ and $t=5000$. Again, we observe that the particles converge to different equilibria, the homogenized system converging to a mean-zero distribution with peaks at 1 and $-\,1$, while for positive values of the parameter $\epsilon$ the system converges to a distribution with $\bar{X}_t=-\,1$. Similarly to the previous case, we superpose the corresponding invariant measure, rescaled for comparison, for this parameter regime on the $t=5000$ snapshot, and again we observe that the empirical density of the system of interacting diffusions converges to the steady-state solution computed by solving the stationary McKean–Vlasov equation, which is also obtained by time evolution of the Fokker–Planck equation (see Figs. 17, 18 in Sect. 4.5).

Finally, we plot in Fig. 16 the average $\bar{X}_t^N$ of the $N=500$ particles for the case of a bistable large-scale potential with nonseparable fluctuations. We observe here that the critical temperature for the homogenized dynamics is different than that for the full dynamics. In particular, the phase transition occurs for $\beta \approx 10.4 > 5.6$ for the homogenized problem, while for finite values of $\epsilon$ there already exist several branches at this value of $\beta$.

### Time-Dependent McKean–Vlasov Evolution

We performed time-dependent simulations of the evolution of the nonlinear McKean–Vlasov equation both for the full and for the homogenized dynamics. We present below the results corresponding to the cases presented for the Monte Carlo simulations.

We recall that, for the case when we take $N\rightarrow \infty$ first while keeping $\epsilon >0$ fixed, the McKean–Vlasov–Fokker–Planck equation that we need to solve is

4.6 $$\begin{aligned} \frac{\partial p}{\partial t}= & {} \frac{\partial }{\partial x}\left( \beta ^{-1}\frac{\partial p}{\partial x} + \partial _x V^\epsilon (x) p + \theta \left( x - \int x p(x,t) \, \hbox {d}x \right) p\right) , \end{aligned}$$

whereas for the case when we first homogenize the dynamics and then pass to the mean field limit the McKean–Vlasov equation becomes

4.7 $$\begin{aligned} \frac{\partial p}{\partial t}= & {} \frac{\partial }{\partial x}\left[ \beta ^{-1}\frac{\partial \left( {\mathcal {M}}(x) p\right) }{\partial x} \!+\! {\mathcal {M}}(x)\left( V'_0(x) +\psi '(x) + \theta \left( x - \int x p(x,t) \, \hbox {d}x \right) \right) p\right. \nonumber \\&\left. + \,\beta ^{-1}\frac{\partial {\mathcal {M}}(x)}{\partial x}p\right] , \end{aligned}$$

with $\psi (x)$ and ${\mathcal {M}}(x)$ given by (2.20) and (2.16), respectively.

To solve the McKean–Vlasov evolution PDE, we used the positivity preserving, entropy decreasing finite volume scheme from Carrillo et al. (2015). We point out that this scheme solves the equations using no-flux boundary conditions. We use these boundary conditions and a sufficiently large domain. We used the same initial conditions for the time-dependent Fokker–Planck simulations as the ones used for the Monte Carlo simulations, i.e., the initial condition was the PDF for a normal distribution with mean zero and variance 4. However, for the bistable large-scale potential with nonseparable fluctuations in the finite but positive $\epsilon$ case—see left panel in Fig. 18—we needed to use a different initial condition: Here we used a normal distribution with mean $-\,0.1$ and variance 4. This is likely because the value of $\beta$ we chose here was close to the bifurcation point and the mean-zero solution was still being picked up on the time evolution.

We present below the results for the case of a convex large-scale potential $V_0^c$ with separable fluctuations—the same case presented in Figs. 11, 12 and 13. The parameters used were $\theta = 2$, $\beta = 8$, $\delta = 1$, $\epsilon = 0.1$.

As expected, the results obtained by solving the time-dependent McKean–Vlasov equation are in agreement with the results obtained from the Monte Carlo simulations and from solving the stationary McKean–Vlasov equation—i.e., the self-consistency equation. We note that, similarly to what we observed in the solution of the system of interacting particles, the solution to the McKean–Vlasov equation converges to its steady state faster for the full dynamics than for the homogenized equation. This observation can be quantified by comparing the convergence rates in the weighted $L^2$ or relative entropy exponential estimates, in particular by comparing the constants in the Poincaré and logarithmic Sobolev inequalities for the full and for the homogenized dynamics. A preliminary study of this—for the Fokker–Planck operator of the finite-dimensional dynamics—was presented in Duncan et al. (2016b).

Finally, we present numerical results for the case of a bistable large-scale potential $V_0^b$ with nonseparable fluctuations—the same case presented in Figs. 14, 15 and 16. The parameters used were $\theta = 0.5$, $\beta \approx 5.6$, $\delta = 1$, $\epsilon = 0.1$.

As expected, the solutions converge to those computed by solving the stationary McKean–Vlasov equation and are qualitatively similar to those obtained from the particle system simulations; see Fig. 15. In this case, the solution of the time-dependent McKean–Vlasov PDE converges to a steady state slower for the full dynamics, in comparison with the homogenized dynamics. We believe that this is related to the phenomenon of critical slowing down (Shiino 1987) when the dynamics is close to a bifurcation, since the inverse temperature $\beta ^{-1}$ that we use for the simulations is close to the critical temperature $\beta _C^{-1}$ for the full dynamics.

## Conclusions and Further Work

The combined mean field and homogenization limit for a system of interacting diffusions in a two-scale confining potential was studied in this paper. In particular, the homogenized McKean–Vlasov equation was obtained and studied and the bifurcation diagram for the stationary states was considered. It was shown, by means of analysis and extensive numerical simulations, that the homogenization and mean field limits, at the level of the bifurcation diagram (i.e., when combined with the long time limit), do not commute for nonseparable two-scale potentials. Furthermore, it was shown that the bifurcation diagrams can be completely different for small but finite $\epsilon$ and for the homogenized McKean–Vlasov equation.

It should be emphasized, as is clearly explained in Chayes and Panferov (2010), see in particular the remarks at the end of Sec. 2 of this paper, that the connection between bifurcations and phase transitions for the McKean–Vlasov dynamics is not entirely straightforward. In particular, in order for a bifurcation point to correspond to a genuine phase transition, it is not sufficient to have the emergence of a new branch of solutions, but these emergent solutions should have a lower free energy. More precisely, it was shown in Chayes and Panferov (2010) for the McKean–Vlasov dynamics on the torus and with a finite-range interaction potential that the loss of linear stability of the uniform state—which corresponds to the mean-zero Gibbs state in our setting—does not imply a second-order phase transition. Furthermore, the critical temperature (or, equivalently, critical interaction strength) at which first-order phase transitions occur is lower than the temperature at which the pitchfork bifurcation happens. For the problem that we studied, supercritical pitchfork bifurcations occur which correspond to second-order (continuous) phase transitions. On the other hand, when only saddle node bifurcations are present, e.g., in Fig. 7b, then the mean-zero solution is still the global minimizer of the free energy; see Fig. 7d. In particular, no first-order phase transitions seem to appear in the McKean–Vlasov model that we studied in this work.

There are many open questions that are not addressed in this work. First, the rigorous multiscale analysis for the McKean–Vlasov equation in locally periodic potentials needs to be carried out. Perhaps more importantly, the rigorous construction of the bifurcation diagram in the presence of infinitely many local minima in the confining potential, thus extending the results presented in, e.g., Dawson (1983), Tamura (1984), Tugaut (2014), appears to be completely open. Furthermore, the study of the stability of stationary solutions to the McKean–Vlasov equation in the presence of a multiscale structure, as well as the analysis of the problem of convergence to equilibrium in this setting is an intriguing question. Finally, the extension of the work presented in this paper to higher dimensions presents additional challenges. We mention, for example, that the corresponding nonlinear diffusion process does not have to be reversible (Lelievre et al. 2013; Duncan et al. 2016). We believe that the results reported in this work open up a new exciting avenue of research in the study of mean field limits for interacting diffusions in the presence of many local minima, with potentially interesting applications to the study of McKean–Vlasov-based mathematical models in the social sciences.

## Footnotes

1 This corresponds to the mean field ansatz for the *N*-particle distribution function, $p_N(x^1, \dots x^N,t ) = \prod _{n=1}^N p(x^n,t)$ and passing to the limit as $N\rightarrow \infty$. See Martzel and Aslangul (2001), Balescu (1997).

2 In fact, in these papers a potential with *N* microscales and one macroscopic scale of the form $V^{\epsilon }(\mathbf{x}) = V\left( \mathbf{x}, \frac{\mathbf{x}}{\epsilon }, \frac{\mathbf{x}}{\epsilon ^2}, \dots \frac{\mathbf{x}}{\epsilon ^N} \right)$, where *V* is periodic in all the microvariables is studied. For the purposes of this work, it is sufficient to consider a potential with two characteristic, widely separated, length scales.

3 In fact, the noise in this SDE can be interpreted in the Klimontovich sense:

$$\begin{aligned} \hbox {d}X_t^i = - {\mathcal {M}}(X_t^i) \partial _{x^i}\Psi '(X_t^1, \dots X_t^N) \, \hbox {d}t + \sqrt{2\beta ^{-1}{\mathcal {M}}(X_t^i)} \circ ^{K} \hbox {d}B_t^i, \end{aligned}$$

where $\circ ^{K}$ denotes the Klimontovich stochastic integral; see Duncan et al. (2016b). In particular, the correction to the Itô integral is $\beta ^{-1}{\mathcal {M}}(X_t^i)$ instead of $\frac{1}{2}{\mathcal {M}}(X_t^i)$ that corresponds to the Stratonovich stochastic integral. See Pavliotis (2014, Sec. 3.2) for details.

4 In fact, these papers consider the more general case, where the diffusion coefficient, $\sigma$, also depends on the empirical measure, $\sigma \left( X_t^i, \frac{1}{N}\sum _{j=1}^N X_t^j\right)$.

5 These are variants of boundedness and Lipschitz continuity assumptions for the drift and diffusion coefficients. The estimates on the homogenized coefficients that are obtained in Abdulle et al. (2017), are sufficient in order to invoke the results of Gärtner (1988), Oelschläger (1984). For the purposes of this paper it is sufficient to pass formally to the mean field limit. The rigorous analysis will be presented elsewhere.

6 In Table 1, we denote by $\chi _A$ the characteristic function of the set *A*.

7 The moment bounds in Arnold et al. (1996) were obtained for confining potentials with no oscillatory terms. However, it can be checked that they are also valid for the class of fluctuating potentials that we consider in this work, and that they provide us with bounds on the moments that uniform in $\epsilon$.

8 Rigorous mathematical construction of the arclength continuation methodology can be found, e.g., in Krauskopf (2007) and Allgower and Georg (1990). Some useful practical aspects of implementing arclength continuation are also given in Dhooge et al. (2006). We use Matlab’s toolboxes to compute the integrals in (2.24) and (2.25) and thus need to solve

$$\begin{aligned} \mathbf {F}([p,m]) = \left[ \begin{array}{c} p - p_\infty (x;\theta ,\beta ,m)\\ m-R(m;\theta ,\beta ) \end{array} \right] = 0, \quad \text { and } \quad \mathbf {G}(\beta ) = \beta - \frac{1}{\theta \int x^2p_\infty (x;\theta ,\beta ,0) \ \hbox {d}x} = 0, \end{aligned}$$

where $p_\infty (x;\theta ,\beta ,m)$ is a stationary solution of the McKean–Vlasov equation. We start the algorithm at a sufficiently large $\beta _0$, i.e., at a sufficiently low temperature for which we have a good initial guess for the value of the order parameter.

## References

[CR1] Abdulle, A., Pavliotis, G.A., Vaes, U.: Spectral methods for multiscale stochastic differential equations. arxiv:1609.05097v1 (2017)

[CR2] Allgower, E.L., Georg, K.: Introduction to Numerical Continuation Methods. Colorado State University (1990)

[CR3] Arnold A, Bonilla LL, Markowich PA (1996). Liapunov functionals and large-time-asymptotics of mean-field nonlinear Fokker–Planck equations. Transp. Theory Stat. Phys..

[CR4] Balescu R (1997). Statistical Dynamics. Matter out of Equilibrium..

[CR5] Binney J, Tremaine S (2008). Galactic Dynamics.

[CR6] Bogachev, V.I., Krylov, N.V., Röckner, M., Shaposhnikov, S.V.: Fokker–Planck–Kolmogorov equations. Mathematical Surveys and Monographs, vol. 207. American Mathematical Society, Providence, RI (2015)

[CR7] Carrillo JA, McCann RJ, Villani C (2006). Contractions in the 2-Wasserstein length space and thermalization of granular media. Arch. Ration. Mech. Anal..

[CR8] Carrillo JA, Chertock A, Huang Y (2015). A finite-volume method for nonlinear nonlocal equations with a gradient flow structure. Commun. Comput. Phys..

[CR9] Chayes L, Panferov V (2010). The McKean–Vlasov equation in finite volume. J. Stat. Phys..

[CR10] Dawson DA (1983). Critical dynamics and fluctuations for a mean-field model of cooperative behavior. J. Stat. Phys..

[CR11] Dhooge, A., Govaerts, W., Kuznetsov, YuA, Mestrom, W., Riet, A.M., Sautois, B.: MATCONT and CL MATCONT: Continuation toolboxes in matlab. Utrecht University, Netherlands and Universiteit Gent, Belgium (2006)

[CR12] Duncan, A.B., Kalliadasis, S., Pavliotis, G.A., Pradas, M.: Noise-induced transitions in rugged energy landscapes. Phys. Rev. E, 94(3), SEP 6 (2016) 10.1103/PhysRevE.94.032107 27739696

[CR13] Duncan, A.B., Pavliotis, G.A.: Brownian motion in an N-scale periodic potential. arXiv:1605.05854 (2016)

[CR14] Duncan AB, Lelievre T, Pavliotis G A (2016). Variance reduction using nonreversible Langevin samplers. J. Stat. Phys..

[CR15] Farkhooi, F., Stannat, W.: A complete mean-field theory for dynamics of binary recurrent neural networks. arXiv:1701.07128v1 (2017) 10.1103/PhysRevLett.119.208301 29219356

[CR16] Frank, T.D.: Nonlinear Fokker–Planck Equations. Springer Series in Synergetics. Springer, Berlin (2005)

[CR17] Garnier J, Papanicolaou G, Yang T-W (2013). Large deviations for a mean field model of systemic risk. SIAM J. Financ. Math..

[CR18] Garnier J, Papanicolaou G, Yang T-W (2017). Consensus convergence with stochastic effects. Vietnam J. Math..

[CR19] Gärtner J (1988). On the McKean–Vlasov limit for interacting diffusions. Math. Nachr..

[CR20] Goddard, B.D., Nold, A., Savva, N., Pavliotis, G.A., Kalliadasis, S.: General dynamical density functional theory for classical fluids. Phys. Rev. Lett. 109(12), SEP 18 (2012a) 10.1103/PhysRevLett.109.120603 23005931

[CR21] Goddard BD, Pavliotis GA, Kalliadasis S (2012). The overdamped limit of dynamic density functional theory: rigorous results. Multiscale Model. Simul..

[CR22] Hartmann C, Latorre JC, Zhang W, Pavliotis GA (2014). Optimal control of multiscale systems using reduced-order models. J. Comput. Dyn..

[CR23] Horsthemke, W., Lefever, R.: Noise-induced transitions, volume 15 of Springer Series in Synergetics. Springer-Verlag, Berlin, 1984. Theory and applications in physics, chemistry, and biology (1984)

[CR24] Imkeller P, Namachchivaya NS, Perkowski N, Yeong HC (2013). Dimensional reduction in nonlinear filtering: a homogenization approach. Ann. Appl. Probab..

[CR25] Krauskopf B (2007). Numerical Continuation Methods for Dynamical Systems.

[CR26] Lelievre T, Nier F, Pavliotis GA (2013). Optimal non-reversible linear drift for the convergence to equilibrium of a diffusion. J. Stat. Phys..

[CR27] Lućon E, Stannat W (2016). Transition from gaussian to non-gaussian fluctuations for mean-field diffusions in spatial interaction. Ann. Probab..

[CR28] Martzel N, Aslangul C (2001). Mean-field treatment of the many-body Fokker-Planck equation. J. Phys. A.

[CR29] McKean HP (1966). A class of Markov processes associated with nonlinear parabolic equations. Proc. Nat. Acad. Sci. USA.

[CR30] McKean, Jr. H.P.: Propagation of chaos for a class of non-linear parabolic equations. In: Stochastic Differential Equations (Lecture Series in Differential Equations, Session 7, Catholic Univ., 1967), pages 41–57. Air Force Office Sci. Res., Arlington, VA (1967)

[CR31] Motsch S, Tadmor E (2014). Heterophilious dynamics enhances consensus. SIAM Rev..

[CR32] Oelschläger K (1984). A martingale approach to the law of large numbers for weakly interacting stochastic processes. Ann. Probab..

[CR33] Papavasiliou, A.: Particle filters for multiscale diffusions. In: Conference Oxford sur les méthodes de Monte Carlo séquentielles, volume 19 of ESAIM Proc., pages 108–114. EDP Sci., Les Ulis (2007)

[CR34] Papavasiliou A, Pavliotis GA, Stuart AM (2009). Maximum likelihood drift estimation for multiscale diffusions. Stoch. Process. Appl..

[CR35] Pavliotis, G.A.: Stochastic processes and applications, volume 60 of Texts in Applied Mathematics. Springer, New York, 2014. Diffusion processes, the Fokker–Planck and Langevin equations (2014)

[CR36] Pavliotis GA, Stuart AM (2007). Parameter estimation for multiscale diffusions. J. Stat. Phys..

[CR37] Pavliotis, G.A., Stuart, A.M.: Multiscale Methods, volume 53 of Texts in Applied Mathematics. Springer, New York, Averaging and Homogenization (2008)

[CR38] Pinnau R, Totzeck C, Tse O, Martin S (2017). A consensus-based model for global optimization and its mean-field limit. Math. Models Methods Appl. Sci..

[CR39] Shiino M (1987). Dynamical behavior of stochastic systems of infinitely many coupled nonlinear oscillators exhibiting phase transitions of mean-field type: H theorem on asymptotic approach to equilibrium and critical slowing down of order-parameter fiuctuations. Phys. Rev. A.

[CR40] Spiliopoulos K (2013). Large deviations and importance sampling for systems of slow-fast motion. Appl. Math. Optim..

[CR41] Tamura Y (1984). On asymptotic behaviors of the solution of a non-linear diffusion equation. J. Fac. Sci. Univ. Tokyo.

[CR42] Tamura Y (1987). Free energy and the convergence of distributions of diffusion processes of McKean type. J. Fac. Sci. Univ. Tokyo Sect. IA Math..

[CR43] Tugaut J (2014). Phase transitions of McKean–Vlasov processes in double-wells landscape. Stochastics.

[CR44] Villani, C.: Topics in optimal transportation. Graduate Studies in Mathematics, vol. 58. American Mathematical Society, Providence, RI (2003)

